# Fumarate and Its Esters: Physiology, Pathology, Molecular Mechanisms, Diseases, and Therapy

**DOI:** 10.1002/mco2.71059

**Published:** 2026-10-11

**Authors:** Peng Zhu, Mengling You, Yi Zeng, Jinglin Zhang, Si Chen, Rong Rong, Xiaobo Xia

**Affiliations:** ^1^ Eye Center of Xiangya Hospital Central South University Changsha Hunan China; ^2^ Hunan Key Laboratory of Ophthalmology Changsha Hunan China; ^3^ National Clinical Key Specialty of Ophthalmology Changsha Hunan China; ^4^ National Clinical Research Center for Geriatric Diseases (Xiangya Hospital) Central South University Changsha Hunan China

**Keywords:** fumarate, fumaric acid ester, immunoregulation, oxidative stress, succination, TCA cycle

## Abstract

Fumarate is a critical intermediate of the tricarboxylic acid cycle that, beyond its metabolic role, has emerged as an important regulator of cellular signaling. Fumaric acid esters (FAEs), including dimethyl fumarate and monomethyl fumarate, have been utilized as therapeutic agents for immune‐mediated diseases and are being explored for broader applications. Accumulating evidence indicates that both fumarate and FAEs modulate diverse biological processes through mechanisms such as protein succination and metabolic reprogramming. In this review, we discuss the current understanding of fumarate and FAEs in health and disease, integrating evidence from various clinical and preclinical research. We conclude that protein succination represents an important mechanism for many of their biological activities, whereas differences in electrophilicity, cell permeability, intracellular distribution, and the metabolic context dictate distinct molecular targets and functional outcomes. We further evaluate the opportunities and limitations of current research, highlighting key technical challenges that complicate mechanistic interpretation and clinical translation. By bridging and differentiating the biology of endogenous fumarate and the pharmacology of FAEs, this review provides a conceptual framework for interpreting existing studies and outlines future directions for developing more precise fumarate‐targeted interventions in diseases.

## Graphical abstract

Fumarate is an endogenous immunometabolite that regulates metabolism, redox homeostasis, and inflammation. Although fumaric acid esters (FAEs) share several molecular targets with fumarate, their distinct physicochemical properties confer unique biological activities and therapeutic applications. Understanding the roles of fumarate and FAEs in physiology and pathology, as well as the current status of disease research, will provide support for the development of future treatment strategies.

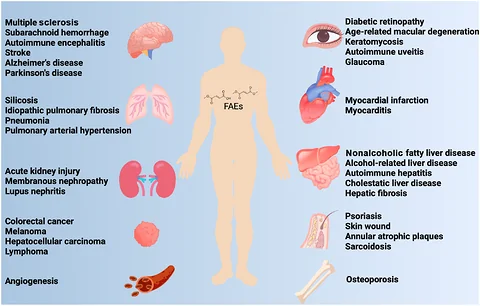

## 1 Introduction

Fumarate, an unsaturated dicarboxylic acid, was first isolated from the plant *Fumaria officinalis* in the early 19th century and later identified as a key intermediate of the tricarboxylic acid (TCA) cycle [1]. Historically recognized as a fundamental metabolic intermediate, fumarate has now emerged as a biologically active metabolite with diverse and context‐dependent roles in human health and disease. It is a significant metabolite within the human body, directly participating in the TCA cycle and essential for maintaining normal physiological functions. Over the past decades, researchers have progressively revealed its intricate functions and multifaceted roles. Previous studies have shown that mutations in the fumarate hydratase (FH) gene could lead to fumarate accumulation and activation of procarcinogenic signaling cascades [2]. Hence, fumarate is frequently characterized as a potential oncometabolite. However, there are also studies suggesting that fumarate possesses cytoprotective properties. For example, in *Fh1* cardiac knockout mice, the accumulation of fumarate markedly alleviates cardiac ischemia–reperfusion (IR) injury (IRI) [3]. Clinically, fumarate derivatives, such as monomethyl fumarate (MMF) and dimethyl fumarate (DMF), are effective in treating relapsing‐remitting multiple sclerosis (RRMS) and psoriasis, with United States Food and Drug Administration approval [1, 4]. This dual role highlights the complexity of fumarate's function in biomedical contexts.

In recent decades, significant efforts have been undertaken to elucidate the biological effects of fumarate. Numerous contemporary studies have delineated its roles in the regulation of immune responses, the mitigation of hypertension, and the reduction of tissue damage, among other functions [5, 6, 7]. It has also been discovered that fumarate modifies the cysteine residues of target proteins through a process known as succination, which is a primary mechanism through which it exerts various effects [8]. Fumarate also acts as a competitive inhibitor of α‐ketoglutarate (α‐KG)‐dependent dioxygenases (α‐KGDD), thereby affecting the activity and function of various enzymes [9]. For example, fumarate primarily mediates tumorigenesis, proliferation, and invasion by influencing signaling pathways such as lysine demethylase 4A/B (KDM4A/B), prolyl hydroxylase (PHD)/hypoxia‐inducible factor 1 (HIF‐1), and zeta‐chain‐associated protein 70 (ZAP70). It also exerts antioxidative and anti‐inflammatory effects through pathways like nuclear factor erythroid‐2‐related factor 2 (NRF2), nuclear factor κ‐light‐chain‐enhancer of activated B cells (NF‐κB), and NOD‐, LRR‐, and pyrin domain‐containing protein 3 (NLRP3), thereby contributing to a range of biological functions. Due to the diverse biological activities of fumarate, researchers are actively exploring its therapeutic potential across various diseases and pursuing corresponding clinical translation efforts (Figure 1). However, only the treatment of psoriasis and multiple sclerosis (MS) has been authorized for fumarate‐related medications in clinical practice to date. Its clinical translation still needs to be actively advanced.

**FIGURE 1:**
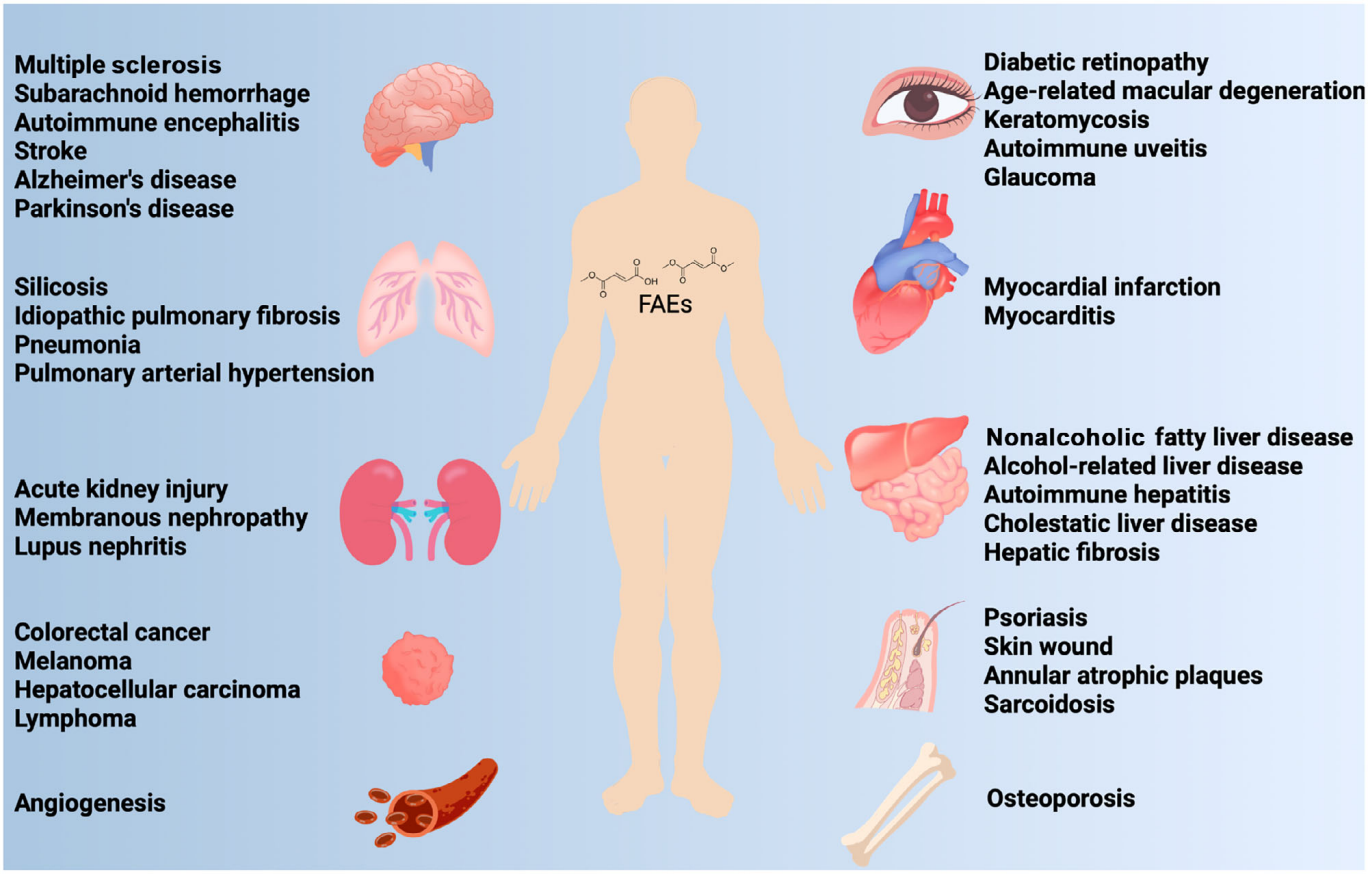
The therapeutic landscape of FAEs across multiple organs and human diseases. FAEs exhibit therapeutic potential across diverse human diseases via their antioxidant, anti‐inflammatory, and immunomodulatory activities. Multiple sclerosis and psoriasis represent clinically approved indications for DMF/MMF therapy.

Given the complexities of fumarate's context‐dependent roles, a systematic and up‐to‐date synthesis of the literature is urgently needed. This review provides a comprehensive overview of metabolism, biological functions, and specific roles in pathophysiological processes and selected diseases of fumarate and fumaric acid esters (FAEs). It also explores the intricate signaling mechanisms involved and discusses related clinical therapeutic strategies. The key highlights of this review are: (1) an integrated analysis of the functions of fumarate and FAEs across different tissues and disease contexts; (2) a critical evaluation of current clinical applications and emerging therapeutic opportunities; and (3) a forward looking discussion of the major challenges (e.g., target specificity, delivery, long term safety) and future research directions needed to accelerate clinical translation. By presenting these insights, we aim to deepen the understanding of fumarate's comprehensive functions in human physiology and disease states and to promote its future clinical translation.

## 2 Metabolism, Biochemistry, and Research Strategy

### 2.1 Fumarate Metabolism

Understanding the generation and clearance of fumarate is fundamental to recognizing its role in both health and disease (Figure 2). The generation of fumarate in the body occurs through multiple pathways, primarily including the TCA cycle, urea cycle, purine nucleotide metabolism, and tyrosine metabolism. Its clearance mostly depends on FH, an enzyme that transforms fumarate into malate. Under normal conditions, the production and clearance of fumarate are kept in relative balance, ensuring the dynamic stability of fumarate levels. This balance is crucial for maintaining cellular energy supply, metabolic processes, and overall human health.

**FIGURE 2:**
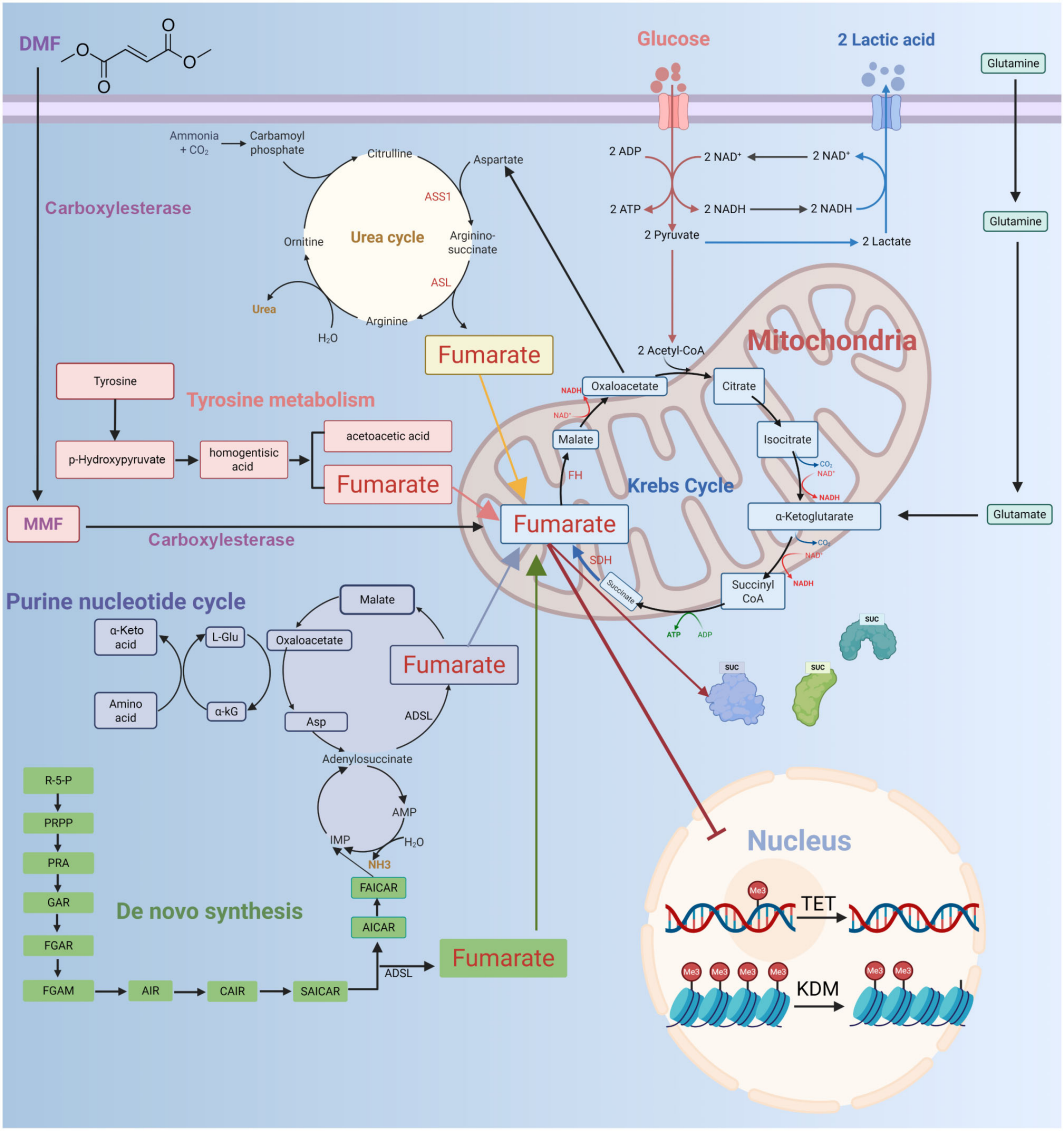
Metabolism of fumarate and FAEs in the body. Fumarate is primarily produced in the TCA cycle, urea cycle, and purine nucleotide metabolism through the catalytic action of specific enzymes on substrates. Factors that enhance these cycles, such as glutamine supplementation, also increase fumarate levels. Fumarate production is also associated with the metabolism of other substances, such as tyrosine. Fumarate clearance is primarily dependent on FH. Free fumarate levels can also be affected by protein modification or by competing with α‐ketoglutarate for binding to the enzyme active site. Upon oral administration, DMF is rapidly hydrolyzed to MMF in the intestine, which can be further hydrolyzed to fumarate. R‐5‐P, ribose‐5‐phosphate; PRPP, 5‐phosphoribosyl‐1‐pyrophsophate; PRA, 5‐phosphoribosylamine; SAICAR, succinyl aminoimidazole carboxamide ribosyl‐5′‐phosphate; AICAR, aminoimidazole carboxamide ribosyl‐5′‐phosphate; ADSL, adenylosuccinate lyase; ASS1, argininosuccinate synthase‐1; ASL, argininosuccinate lyase.

Fumarate in the mitochondria primarily originates from the TCA cycle, with succinate dehydrogenase (SDH) playing a key role. In the TCA cycle, α‐KG undergoes decarboxylation to form succinyl‐CoA, which is further hydrolyzed to succinate. Succinate is then converted to fumarate by SDH, which is also a component of the respiratory chain. The factors that can cause the increase in TCA cycle flux may lead to an increase in fumarate level. For instance, glutamine that transported into the cell via amino acid transporters can be converted into glutamate, which is further transformed into α‐KG, thus replenishing the TCA cycle and increasing fumarate production. This process is of significant importance in trained immunity induction and cancer cell metabolism [10, 11]. Under normal circumstances, fumarate levels in the mitochondria are predominantly regulated by SDH and FH. FH is also expressed in the cytoplasm and nucleus [12, 13], where it can similarly catalyze the reversible hydration/dehydration reaction between fumarate and malate.

The urea cycle is another significant metabolic pathway in the human body, essential for the removal of excess nitrogen and detoxification. The urea cycle enzyme argininosuccinate synthase‐1 (ASS1) couples nitrogen from aspartate and citrulline to produce argininosuccinate. Subsequently, argininosuccinate is cleaved by argininosuccinate lyase (ASL) into fumarate and l‐arginine. Fumarate subsequently enters the TCA cycle for further metabolism, while arginine is further cleaved in the urea cycle to produce ornithine and urea [14]. The urea cycle primarily occurs in the liver, and this process not only eliminates harmful substances but also produces fumarate, which contributes to providing sufficient energy for the liver. Under normal conditions, ASL is expressed in various tissues, including the brain, kidney, lung, and ocular tissues, as well as in macrophages and vascular smooth muscle cells. Although its expression is not as abundant as in the liver, it is sufficient to indicate that fumarate can also be produced through this pathway in various tissues or cells. In other words, beyond the liver, ASS1 and ASL collaboratively engage in the cytoplasmic arginine–citrulline cycle, facilitating the cellular requirement for fumarate. Interestingly, following exposure to doxorubicin, which induces DNA damage, the levels of fumarate in the nucleus increase. This elevation is attributed to enhanced interaction between ASS1 and Importin‐7, which facilitates the entry of ASS1 into the nucleus [14]. In lipopolysaccharide (LPS)‐activated macrophages, cytoplasmic fumarate levels also increase, which is attributed to the enhanced flux from aspartate to argininosuccinate and increased expression of ASS1 [15]. Notably, FH levels decrease following long‐term treatment of LPS, contributing to the accumulation of fumarate.

Fumarate is also produced during purine nucleotide metabolism. The de novo synthesis of purine nucleotides requires the initial synthesis of inosine monophosphate (IMP), which is then converted into adenosine monophosphate (AMP) and guanosine monophosphate. During IMP synthesis, fumarate is produced as a byproduct, catalyzed by adenylosuccinate lyase (ADSL) [16]. Adenylosuccinate synthetase catalyzes the condensation of IMP with aspartic acid to form adenylosuccinate, which is subsequently cleaved into AMP and fumarate by ADSL [16]. Notably, the aspartate required for the reaction can be generated through transamination, where oxaloacetate accepts the amino group from other amino acids. This transamination reaction is the primary pathway for amino acid deamination in skeletal and cardiac muscle and is closely related to the interaction between amino acids and the TCA cycle.

### 2.2 DMF and MMF Metabolism

Currently, DMF and MMF are often used as supplements of fumarate in research [17]. Chemically, the presence of ester groups and an activated double bond constitute the two primary features of DMF and MMF. Although DMF and MMF differ by only a single methyl group in their chemical structures, DMF exhibits a substantially higher hydrolysis rate and greater plasma membrane permeability [18, 19], which makes their metabolic characteristics and roles different in vivo and in vitro. Upon oral administration, DMF is rapidly hydrolyzed to MMF, which has a longer half‐life and reaches peak plasma concentrations after 5–6 h. MMF is metabolized through the TCA cycle and primarily excreted via respiration [20]. The metabolic characteristics of DMF after administration suggest that it may act only as a prodrug. It has been shown that there is no detectable DMF in plasma after taking DMF [21]. A study in rats indicates that it is highly improbable for DMF to exit the intestinal mucosa before metabolism, providing evidence for its rapid metabolism [22]. Previous studies revealed that although DMF may bind to glutathione (GSH) to form adducts [22, 23], the great majority of orally administered DMF undergoes spontaneous or esterase‐mediated hydrolysis in the intestinal tract before reaching the systemic circulation. In cell experiments or even local administration, the hydrolysis rate of DMF is relatively slow. As more DMF enters the cells, the effects produced are also different, which will be discussed in later sections.

### 2.3 Biochemistry of Fumarate and FAEs

Esterification significantly alters the physicochemical properties and bioactivity of fumarate. Here, we primarily discuss the biochemical process of fumarate and FAEs in the body and the similarities and differences in chemical properties among fumarate, MMF, and DMF.

Due to its structural similarity to α‐KG, fumarate can inhibit the activity of α‐KGDD. Several dozen α‐KGDDs have been reported in mammalian cells [24], including the JmjC domain‐containing lysine demethylase (KDMs), ten‐eleven translocation (TET) family of DNA hydroxylases, and PHDs. The competitive inhibitory effect on KDMs and TET implies that fumarate has a potential epigenetic regulatory role (Figure 2). Taking PHDs as an example, fumarate has a stronger inhibitory effect than other α‐KG analogs such as succinate or oxaloacetate [25], and the presence of a carbon–carbon double bond may be one of the reasons [26]. In addition, FAEs do not seem to directly inhibit PHDs, because it does not inhibit purified PHDs at a high concentration (10 mM), so their effect in cells can be attributed to the fumarate produced by hydrolysis [25]. We speculate that this is caused by increased steric hindrance and the loss of the carboxyl group.

In terms of polarity, DMF has the weakest polarity, which means that it is more lipid soluble and can pass through cell membranes more easily, but it is also easily hydrolyzed in the body. In terms of electrophilicity and bioreactivity, DMF is the strongest, followed by MMF. The electrophilicity of fumarate and FAEs is the basis for their Michael addition reaction with GSH and cysteine sulfhydryl groups, which is the key mechanism for their antioxidant and other effects (Figure 3).

**FIGURE 3:**
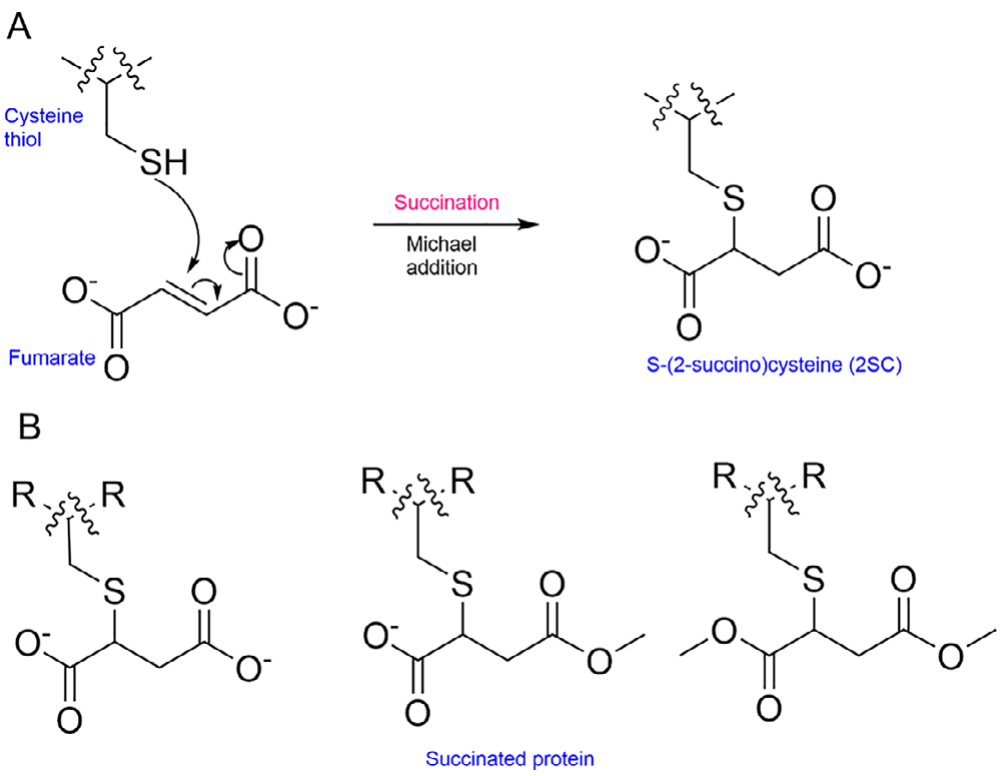
Succination is a chemical reaction between fumarate/FAEs and the thiol group of cysteine. (A) Schematic diagram of the Michael addition reaction between fumarate and cysteine thiol. (B) The chemical structure of proteins modified with fumarate, MMF, and DMF.

The sulfhydryl group (─SH) is a critical component of certain proteins, known for its strong reducing capability. These groups often form part of the active sites of enzymes and functional proteins. Chemically, due to the lone pairs of electrons on the sulfur atom, sulfhydryl groups exhibit nucleophilicity and can react with an electrophilic agent. The main sources of sulfhydryl groups in the body include GSH and cysteine. GSH, a tripeptide containing a cysteine residue, can be modified by fumarate and DMF/MMF [27]. Fumarate can undergo a Michael addition reaction with protein cysteine residues to form S‐(2‐succinyl)cysteine (2SC) [8, 17, 28]. Similarly, DMF and MMF can modify protein residues, resulting in 2‐dimethyl succinyl‐cysteine or 2‐monomethyl succinyl‐cysteine, respectively [17, 29]. However, some studies have suggested that fumarate itself is nearly inert in this reaction and only becomes active after protonation [23, 30], which may explain the phenomenon of protein succination observed in hereditary leiomyomatosis and renal cell cancer (HLRCC), as fumarate and lactate levels are significantly elevated. It is important to note that under physiological conditions, certain proteins can undergo succination, such as albumin and skin collagen [8]. This suggests that although fumarate is less active as an electron acceptor in the Michael addition reaction compared with DMF/MMF, succination can still occur under physiological conditions. In other scenarios, such as when the glucose level is high, fumarate levels also increase significantly, accompanied by an increase in protein succination [31, 32]. Additionally, the results of in vitro incubation of fumarate with glyceraldehyde‐3‐phosphate dehydrogenase (GAPDH), as well as the succination of GAPDH observed in healthy mice and humans, demonstrate its ability to modify and inhibit the latter [28, 33].

To date, a series of succination‐sensitive proteins have been identified, such as Kelch‐like ECH‐associated protein 1 (KEAP1), GAPDH [28], gasdermin D (GSDMD) [29], IκB kinase (IKK) complex [34, 35], and phosphatase and tensin homolog (PTEN) [17]. Table 1 summarizes the proteins known to be modified by fumarate or FAEs and their respective functions. There is no doubt that due to the differing reactivity of the three compounds, the modification of different targets and different sites on the same target, as well as the effects, will vary. However, due to the rapid hydrolysis of DMF after oral administration, this may primarily manifest in cellular experiments. Proteomics studies have revealed that several proteins sensitive to DMF covalent modification are involved in regulating T cell function, including protein kinase Cθ, whereas MMF does not exhibit this effect [19]. DMF and MMF also exert distinct modification effects on the same protein; for example, in KEAP1, modification of cysteine residues such as Cys151, Cys257, and Cys273 contributes to NRF2 stabilization. In HEK293FT cells, there were significant differences in the modification rates of the Cys151 site of KEAP1 at the same concentrations of DMF and MMF; this difference was even more pronounced at Cys257 and Cys273. This differential modification can explain why there are differences in the extent of NRF2 nuclear translocation and the expression of downstream target genes between the two treated cells [36]. It is also worth noting that DMF causes acute concentration‐dependent depletion of GSH in vitro, followed by a rapid recovery of GSH levels, while MMF does not cause acute depletion of GSH, but its GSH accumulation effect is also weaker [36].

**TABLE 1:** The primary proteins that undergo succination modification.

Targets	Electrophiles	Major sites	Role	Effects resulting from modifications	References
Glutathione (GSH, tripeptide)	Fumarate, MMF, DMF	Cys	Antioxidant	Elevates reactive oxygen species (ROS) levels and activates the NRF2 signaling pathway	[37, 38, 39]
Glyceraldehyde‑3‑phosphate dehydrogenase (GAPDH)	Fumarate, MMF, DMF	Cys149, 244, 152	The key enzyme in glycolysis	Glycolytic disruption	[28, 33, 40]
Kelch‐like ECH‐associated protein 1 (KEAP1)	Fumarate, MMF, DMF	Cys151	The sensor of oxidant and electrophilic reagent	Activates NRF2 transcription activity	[41, 42]
Gasdermin D (GSDMD)	Fumarate, MMF, DMF	Cys191	The executor of pyroptosis	Blocks the formation of GSDMD‐N and cell death	[29]
Gasdermin E (GSDME)	Fumarate, MMF, DMF	Cys45	The executor of pyroptosis	Attenuates the generation of GSDME‐N and cell death	[29]
Zeta‐chain‐associated protein 70 (ZAP70)	Fumarate, MMF, DMF	Cys96 and Cys102	A tyrosine kinase that plays a critical role in TCR signaling	Impairs TCR signaling and T cell responses	[43]
Glutathione peroxidase 4 (GPX4)^a^	Fumarate, MMF, DMF	Cys93	The primary cellular defender against ferroptosis	Represses GPX4 activity, enhancing ferroptosis sensitivity	[44]
Phosphatase and tensin homolog (PTEN)	Fumarate	Cys211	Tumor suppressor	Activates PI3K/AKT signaling and promotes tumorigenesis	[17]
Inhibitor of κB kinase β (IKKβ)	DMF	Cys179	The key regulator of the NF‐κB signaling pathway	Disrupts the activation of the IKK complex, leading to reduced nuclear translocation of RelA/p65	[34]
RelA/p65	DMF	Cys38	A constituent element of the NF‐κB complex	Blocks p65 nuclear translocation and DNA binding activity	[45]
Ribosomal S6 kinase 2 (RSK2)^b^	DMF	Cys599	An important signal transduction kinase	Inhibits the activation of RSK2 and thus the phosphorylation of RelA/p65	[46, 47]
Mitogen and stress‐activated kinases 1 (MSK1)^b^	DMF	Cys603	Involved in gene transcriptional regulation and epigenetic regulation	Inhibits the activation of MSK1 and thus the phosphorylation of RelA/p65	[47]

*Notes*: The electrophiles listed in the table are well supported by clear evidence.

Abbreviations: NRF2, nuclear factor erythroid‐2‐related factor 2; TCR, T‑cell receptor; PI3K, phosphatidylinositol 3‑kinase; AKT, protein kinase B.

^a^ DMF and MMF could increase GPX4 levels, which may be related to the activation of the NRF2 pathway [48].

^b^ It was shown that only DMF, but not MMF, could covalently react with cysteine residues in the C‐terminal kinase domain of RSK2 and MSK1 [47].

### 2.4 Research Strategy

Manipulating intracellular fumarate levels is central to mechanistic investigations, which is currently achieved primarily through four major strategies as shown in Table 2: FAEs supplementation (e.g., DMF/MMF), *FH* gene knockout (e.g., via the CRISPR–Cas9 system or tamoxifen induction), pharmacological FH inhibition (e.g., using FHIN1), or the induction of endogenous metabolic stress (e.g., LPS stimulation of macrophages). Although each of these methods successfully elevates intracellular fumarate levels, their mechanistic biological consequences exhibit pronounced differences. While FAE supplementation increases fumarate content, the resultant phenotypic effects may actually be driven by the highly reactive parental molecules, DMF and MMF, necessitating cautious differentiation; nevertheless, this strategy remains undoubtedly the optimal choice for interrogating the therapeutic efficacy and underlying mechanisms of FAEs. Conversely, FH knockout models achieve a dramatic accumulation of fumarate but are intrinsically accompanied by a structural disruption of the TCA cycle. Research has demonstrated that in *Fh1* ^−/−^ renal epithelial cells, fumarate levels can surge by over 100‐fold, concurrent with an elevation in succinate and marked reductions in malate and citrate [49]. Notably, rather than simply succumbing to catastrophic metabolic collapse, these cells undergo survival adaptation by establishing alternative metabolic shunts. Specifically, glutamine‐derived carbon flux is redirected toward the heme synthesis and degradation pathway, ultimately being excreted as bilirubin. This rewiring not only alleviates the toxic accumulation of TCA intermediates but also partially sustains mitochondrial NADH production capabilities [49]. This metabolic reprogramming not only unveils the survival strategy of FH‐deficient cells but also engenders a distinct metabolic dependency. Consequently, this renders heme oxygenase a potential synthetic lethal target, providing a novel theoretical foundation for the precision treatment of HLRCC. However, variations in mitochondrial membrane potential within FH‐deficient renal epithelial cells remain a subject of debate. Despite utilizing the same potentiometric probe, tetramethyl rhodamine ethyl ester, for detection, independent studies have paradoxically reported both normal and significantly elevated mitochondrial membrane potentials [49, 50]. This divergence may stem from multilayered metabolic compensatory mechanisms established during long‐term FH deficiency—including the downregulation of mitochondrial respiratory chain complex activities, enhanced autophagy, and the remodeling of the antioxidant defense system—which collectively culminate in a dynamic resetting of the mitochondrial membrane potential under differing experimental conditions. Alternatively, it may be associated with the generation and tissue origin of the specific cell lines used. In contrast, within macrophages, an elevation in mitochondrial membrane potential is consistently observed—accompanied by enhanced production and release of interferon (IFN)‐β—whether induced by prolonged LPS‐stimulated FH inhibition, pharmacological inhibition with FHIN1, or tamoxifen‐induced conditional *Fh1* knockout [15]. This suggests that fumarate accumulation in macrophages primarily reflects an acute or subacute inflammatory stress state, wherein mitochondrial functional remodeling is tightly coupled with immune activation. Notably, treatment with low concentrations of DMF does not disrupt the basal respiration of the mitochondrial electron transport chain in macrophages, nor does it significantly impair ATP production, redox homeostasis, or mitochondrial membrane potential [15]. This insight indicates that FAEs may exert their immunomodulatory functions through selective signaling modifications rather than via a global perturbation of mitochondrial bioenergetics.

**TABLE 2:** Current strategies for modulating fumarate levels and their distinct characteristics.

Strategy	Background	Applications	Limitations	References
FAEs supplementation	Exogenous supplementation of FAEs with high cell permeability and electrophilicity allows for rapid cellular entry independently of the TCA cycle or other metabolic pathway alterations.	Investigating FAEs pharmacology, protein succination, KEAP1‐NRF2 signaling, and immunomodulation; the primary approach for assessing therapeutic potential	Cannot fully replicate natural fumarate accumulation	[51, 52, 53]
*FH* gene knockout	Permanent/conditional fumarate hydratase (FH) deletion via CRISPR–Cas9 or Cre‐loxP, driving massive endogenous fumarate accumulation, structural TCA cycle disruption, and chronic metabolic reprogramming.	Investigating metabolic adaptation to chronic FH loss, hereditary leiomyomatosis and renal cell cancer (HLRCC) pathogenesis, oncogenic metabolic reprogramming, and synthetic lethal targets	Difficult to decouple the direct actions of fumarate from secondary effects driven by chronic metabolic compensation, transcriptional rewiring, and TCA cycle disruption	[5, 49]
Pharmacological FH inhibition	Transient pharmacological inhibition of FH activity, rapidly elevating endogenous fumarate levels without altering FH protein expression.	Elucidating immediate molecular events and direct signaling triggered by acute fumarate surges, while minimizing long‐term metabolic compensation	Achieves less pronounced fumarate accumulation compared to knock‑out (KO) models, with potential confounding off‐target effects	[15, 54]
Induction of metabolic stress	Inflammatory stimuli induce metabolic reprogramming in immune cells, secondarily suppressing FH activity and promoting endogenous fumarate accumulation.	Investigating immunometabolism, inflammatory signaling, and the biological functions of fumarate as an immunometabolite	Hard to isolate specific fumarate effects from the broader inflammatory cascade and other lipopolysaccharide (LPS)‐induced signaling pathways	[15]

In summary, although elevations in fumarate from diverse sources exhibit consistency at the molecular outcome level, the specific metabolic contexts, durations, and cell types involved dictate pronounced differences in their downstream biological effects. Therefore, future investigations dissecting fumarate biology and the underlying mechanisms of FAEs must systematically differentiate and judiciously select among distinct experimental models—namely, genetic deficiency‐induced chronic fumarate accumulation, pharmacological inhibition‐mediated acute fumarate elevation, inflammation‐induced metabolic reprogramming, and exogenous supplementation. Such rigorous discernment is essential to avoid the simplistic extrapolation of mechanistic findings and to provide a more robust theoretical foundation for precision intervention strategies in associated disorders, including HLRCC, inflammatory diseases, and neurodegenerative conditions.

In conclusion, starting from the generation, metabolism, and clearance pathways of fumarate, DMF, and MMF in the human body, this section comprehensively elaborates on the chemical properties of the three substances and their specific biochemical reactions in the biological system. This lays a theoretical foundation for understanding the relationship between the three substances and for fully understanding the role and function of fumarate and FAEs in physiological and pathological processes. At the same time, it can help better understand the medicinal value of fumarate, DMF, and MMF. Understanding the strategy of regulating fumarate level can help to study the pathogenesis and explore the mechanisms related to curative effects.

## 3 Roles of Fumarates and FAEs in Physiological and Pathological Processes

Fumarate and FAEs play important roles in physiological and pathological processes. At the molecular level, they specifically modify protein cysteine residues through the Michael addition reaction, forming stable covalent complexes, thereby changing the conformation and function of target proteins; or as competitive inhibitors of α‐KG, they can bind with high affinity to the active center of α‐KGDD and block related signaling pathways. These molecular interactions can trigger profound cellular biological effects, including but not limited to: significantly reshaping the cellular energy metabolism network and regulating mitochondrial biogenesis. Notably, the biological effects of fumarate and FAEs are related to cell state and type, showing significant tissue specificity and microenvironment dependence. This characteristic enables them to play an important role in maintaining physiological homeostasis and intervening in pathological processes (Figure 4).

**FIGURE 4:**
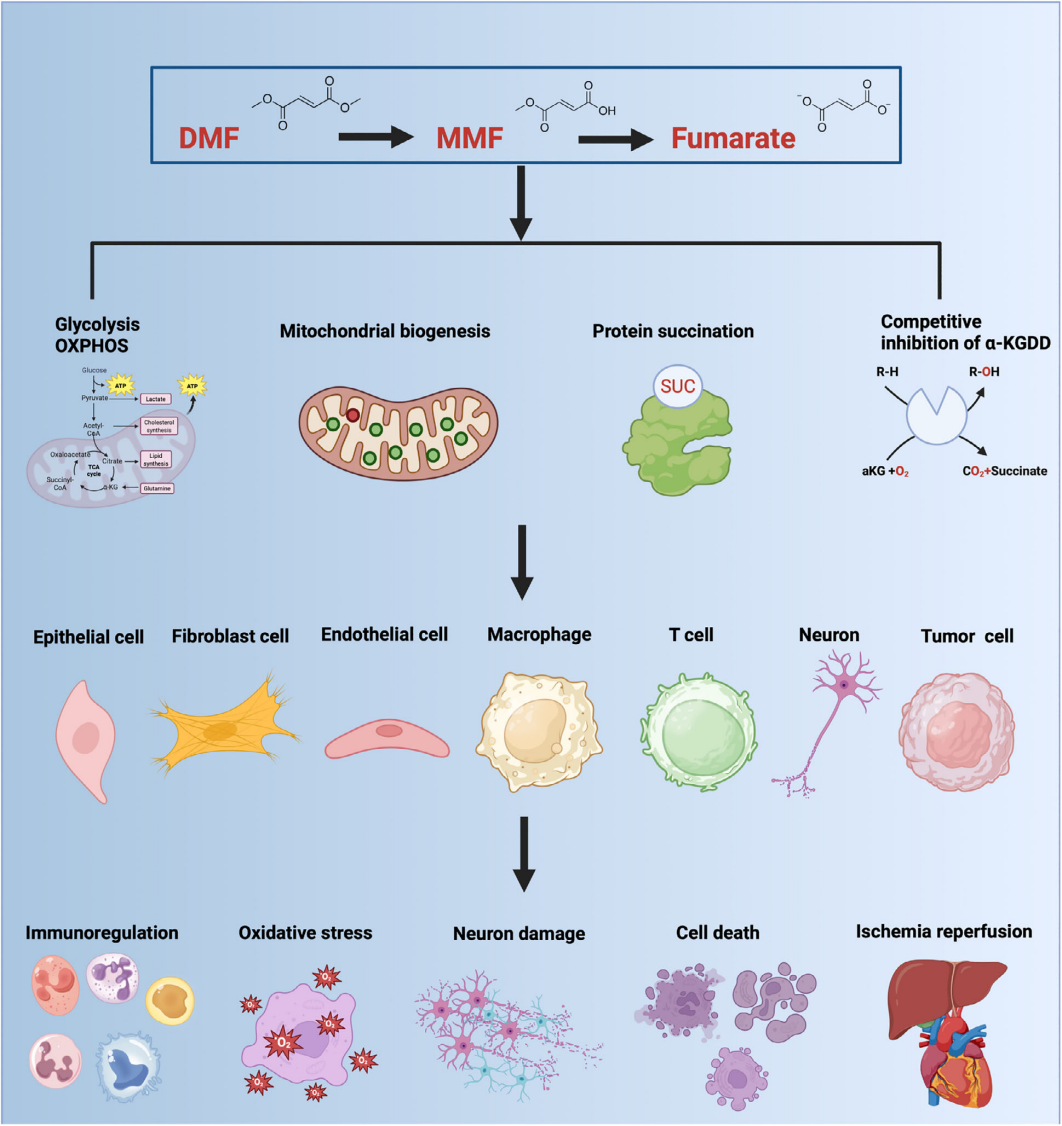
Fumarate and FAEs are involved in the regulation of cellular physiological and pathological processes. Fumarate and FAEs participate in biological processes such as energy metabolism, mitochondrial biogenesis, protein post‐translational modification, and enzymatic activity regulation, thereby widely affecting the activities of diverse cell types, and ultimately modulating critical biological functions and outcomes such as oxidative stress, immune regulation, and cell death.

### 3.1 Molecular Regulation of Protein Function

The essence of fumarate‐induced protein succination lies in the Michael addition reaction between α,β‐unsaturated carbonyl compounds and protein thiol groups. Chemically, fumarate is an α,β‐unsaturated dicarboxylic acid; the carbon–carbon double bond conjugated with its adjacent carbonyl groups constitutes a canonical electrophilic Michael acceptor motif. This structural feature endows fumarate with the capacity to undergo covalent addition with nucleophilic protein residues.

Under physiological or relevant reaction conditions, the sulfhydryl group of cysteine residues in proteins exhibits strong nucleophilicity, which selectively attacks the electron‐deficient double bond of fumarate via Michael addition. This process covalently attaches a fumaryl moiety to the thiol group, generating a stable thioether adduct, namely, 2SC. This specific, irreversible covalent modification targeting cysteine thiols is defined as protein succination.

On this basis, it is noteworthy that molecules with distinct origins or structures display drastically divergent potency in triggering succination, which is predominantly reflected in the comparison among DMF, MMF, and endogenous fumarate. First, with regard to electrophilicity: as a potent Michael acceptor, DMF generally exhibits higher reactivity than MMF and endogenous fumarate. By contrast, the polarizing effect of sulfur atoms confers MMF distinct direct reactivity profiles relative to fumarate. Second, concerning cellular permeability: both DMF and MMF are small hydrophobic molecules with prominent lipophilicity, enabling efficient transmembrane delivery into intracellular compartments. In comparison, endogenous fumarate predominantly exists as negatively charged fumarate anions at physiological pH, resulting in poor membrane permeability; its biological activity thus relies on dedicated transporters or localized high‐concentration accumulation. Third, in terms of intracellular hydrolytic kinetics: acting as a prodrug, DMF undergoes rapid hydrolysis catalyzed by cellular esterases upon cell entry to yield the active intermediate MMF, which further liberates fumarate to drive rapid and robust formation of 2SC. MMF displays a relatively slow hydrolysis rate and weaker succination‐inducing efficiency. Endogenous fumarate, generated during the TCA cycle, possesses a short half‐life and is tightly regulated by metabolic pathways; it only mediates robust protein succination under conditions of transient local hyperconcentration.

In summary, fumarate mediates specific succination modification on cysteine residues through α,β‐unsaturated carbonyl‐driven Michael addition. The discrepancies in electrophilicity, cellular permeability, and intracellular hydrolytic kinetics across DMF, MMF, and endogenous fumarate collectively account for their divergent capacities to elicit protein succination.

Consequently, the protein modification profiles of fumarate, DMF, and MMF are unlikely to be identical, a distinction that is crucial for elucidating disease pathogenesis and clarifying therapeutic mechanisms. However, comprehensively mapping their precise molecular targets remains challenging due to their broad reactivity and complex metabolism in vivo. Current methodologies for investigating fumarate‐ or FAE‐induced protein modifications primarily encompass anti‐2SC antibody detection, mass spectrometry‐based proteomics, and clickable chemical probes; understanding the principles, strengths, and limitations of each approach is fundamental to rationally selecting the appropriate tools for succination research.

Fundamentally, protein succination is characterized by the generation of 2SC. Anti‐2SC antibody detection serves as an indispensable tool for investigating succination, particularly under conditions of elevated endogenous fumarate levels. However, in other pathophysiological states, 2SC accumulation does not reach the exceptionally high levels observed in HLRCC, rendering the sensitivity of anti‐2SC antibody detection potentially insufficient. Furthermore, recent studies have revealed that anti‐2SC antibodies fail to distinguish between 2SC and 2,3‐dicarboxypropylcysteine (2,3‐DCP), two chemically akin structures, where the latter is formed through the reaction of itaconate with protein cysteine residues [55]. To detect low‐abundance 2SC, Liu et al. developed a highly efficient quantitative method based on ethanol/acetyl chloride chemical derivatization [56]. This strategy esterifies the carboxylic acid groups of 2SC to enhance its hydrophobicity and, consequently, its ionization efficiency, effectively pushing the detection sensitivity down to the femtomole level. Crucially, this esterification not only markedly improves the volatility and mass spectrometry ionization efficiency of the analyte, but also amplifies the subtle differences in steric hindrance and hydrophobicity between 2SC and 2,3‐DCP, thereby enabling their effective discrimination [55]. Despite these limitations, the anti‐2SC antibody retains distinct advantages in succination research, as it allows for the visualization of the intracellular spatial distribution of succination [57]. It remains highly suitable for rapid sample screening and the analysis of specific proteins or protein‐protein interactions, serving as a reliable qualitative and quantitative tool in experimental systems where itaconate interference can be excluded.

Zhang et al. designed and synthesized “clickable” fumarate molecular probes by introducing a small, chemically inert alkyne tag onto DMF and MMF. Utilizing the copper‐catalyzed azide–alkyne cycloaddition reaction, they successfully achieved target capture and visualization [58]. Furthermore, by coupling this approach with tandem mass tag‐based quantitative mass spectrometry, a high‐confidence target landscape of fumarates was mapped across the whole proteome. The results revealed that DMF targets are not necessarily regulated by MMF; conversely, a substantial portion (though not all) of MMF targets can also be labeled by DMF, a discrepancy fundamentally dictated by the differences in their chemical reactivity [58]. Although click chemistry probes offer an excellent strategy for identifying the targets of DMF, MMF, and even fumarate, as well as for elucidating and differentiating their underlying mechanisms of action, it is undeniable that the introduction of an alkyne group subtly alters the spatial conformation, hydrophobicity, and local charge distribution of these molecules. Despite the alkyne group being one of the smallest chemical tags available, these alterations may slightly perturb the affinity of the probes for certain proteins, potentially leading to false‐positive or false‐negative results. Additionally, due to the inherent characteristics of click chemistry, such probes are better suited for cell lysates or live‐cell labeling followed by brief lysis, rather than real‐time dynamic tracking in live animals; this limitation arises because the copper ions required for the reaction exhibit severe cytotoxicity and induce the generation of massive amounts of reactive oxygen species (ROS). Furthermore, while this methodology can unequivocally pinpoint which cysteine residues across the proteome are modified by fumarate, it cannot directly distinguish whether a given modification represents a functional active site or a nonfunctional bystander site. Consequently, whether protein function is activated, inhibited, or unaffected postmodification remains to be elucidated through alternative experimental approaches.

### 3.2 Energy Metabolism

Cellular energy metabolism mainly involves glycolysis and oxidative phosphorylation. One of the physiological significances of glycolysis is the rapid supply of energy to cells in hypoxic or hypoxic environments. In fact, in certain cell types, such as tumor cells, astrocytes, and activated lymphocytes, the majority of energy is often derived from glycolysis, even in the presence of sufficient oxygen [28, 59]. Recent studies have highlighted the crucial role of fumarate in the regulation of glycolysis and oxidative phosphorylation [28]. Taking HLRCC, which is closely associated with FH deficiency, as an example, accumulation of fumarate leads to the inhibition of PHD activity and elevated levels of HIF‐1, which shifts metabolic flux toward the glycolytic pathway while inhibiting oxidative phosphorylation [60, 61]. Additionally, recent findings indicate that the accumulation of fumarate alters mitochondrial DNA (mtDNA), causing the loss of respiratory chain components [62]. However, in FH‐mutant tumors, succination of GAPDH was also observed [63], indicating that fumarate may inhibit glycolysis. We propose that FH deficiency inevitably leads to TCA cycle dysfunction, and although accumulated fumarate has a stronger ability to modify GAPDH in an acidic environment, its competitive inhibition of PHDs and the ensuing HIF‐1‐driven metabolic reprogramming may mask the effects of GAPDH succination.

It is important to note that in some cases, FAEs could exhibit glycolytic inhibitory effects. It was found that the lactate production of mouse peritoneal macrophages cotreated with LPS and DMF/MMF was significantly lower than that of cells stimulated with LPS alone, indicating that glycolysis of activated macrophages was inhibited [28]. Kornberg and colleagues demonstrated that modification of GAPDH is critical for this effect [28]. Treating macrophages solely with DMF/MMF does not inhibit glycolysis, which suggests that DMF/MMF may play a potential role in immune regulation. Although fumarate can also modify the cysteine residues of GAPDH, its effect is far less than that of DMF/MMF. We believe that in this case, it is mainly MMF or DMF that plays a role, and the fumarate produced by hydrolysis will smoothly enter the TCA cycle and be metabolized, and its activation effect on the HIF‐1 pathway is not as obvious as when FH is deficient.

In summary, the effects of fumarate and FAEs on glycolysis and oxidative phosphorylation depend on their own reactivity with target proteins, as well as cell type and state. This feature can be a starting point for the treatment of related diseases.

### 3.3 Mitochondrial Function

Mitochondria are indispensable organelles that function not only as cellular powerhouses but also play pivotal roles in maintaining redox homeostasis, regulating apoptosis, and determining cell fate.

Fumarate functions as a metabolic signal within mitochondria, promoting mitochondrial biogenesis [51, 64]. Mitochondrial biogenesis refers to the regenerative program that maintains both the quantity and biomass of mitochondria within cells. Fumarate can enhance the mitochondrial dNTP pool, providing substrates for mtDNA synthesis, and upregulate mtDNA‐encoded proteins [51]. Mechanistically, fumarate promotes mitochondrial biogenesis via NRF2 activation [64], with a more pronounced effect through the malic enzyme 2 (ME2) pathway [51]. It enhances ME2 dimerization, boosting its interaction with dUTPase to elevate mitochondrial dNTP levels and mtDNA replication. Additionally, fumarate disrupts monomeric ME2's binding to mitochondrial ribosomal protein L45, facilitating L45's localization to the inner membrane and promoting ribosome assembly [51].

Fumarate can rescue mitochondrial damage and dysfunction. Cholesterol oxidation products, such as oxysterols like 7‐ketocholesterol and 7β‐hydroxycholesterol, could induce oxidative stress, organelle dysfunction, and cell death [65, 66]. In 158N oligodendrocytes treated with 7‐ketocholesterol, a marked reduction in mitochondrial membrane potential and impaired oxidative phosphorylation were observed [67]. Specifically, 7‐ketocholesterol led to mitochondrial dysfunction in 158N cells, evidenced by an increase in intracellular lactate levels and a significant reduction in TCA cycle intermediates, including fumarate. Studies have shown that treatment with DMF or MMF significantly rescues mitochondrial damage and alleviates the toxic effects of oxysterols on 158N cells.

However, high concentrations of fumarate can induce changes in mitochondrial morphology and lead to the release of its contents, such as mtDNA and mtRNA [5, 15]. It is well established that cytosolic mtDNA can activate innate immune responses and trigger inflammatory reactions [68, 69]. A recent study identified fumarate accumulation as a factor inducing mtDNA release. In addition, they observed mitochondrial morphological changes during this process [5]. Cytosolic exposure of mtDNA may play a pivotal role in maintaining the inflammatory microenvironment observed in HLRCC [5, 69].

### 3.4 Redox Homeostasis

Redox reactions refer to electron transfer reactions between chemical substances and are central to the existence of life. Redox homeostasis refers to the maintenance of balance between oxidants and antioxidants. Excessive oxidants can lead to oxidative distress, while insufficient oxidants can impair signal transduction processes and result in reductive distress [70]. Redox eustress refers to oxidant exposure levels within physiological concentration ranges, which are critical for cellular adaptation, survival, and functional regulation. KEAP1 is a sensor for oxidants and electrophiles and also acts as a negative regulator of NRF2. Increased oxidant levels induce oxidation of KEAP1 cysteine residues, leading to reduced NRF2 degradation and increased nuclear translocation, thereby enhancing GSH biosynthesis and the expression of a series of antioxidant genes. Similarly, the modification of KEAP1 cysteine residues by electrophiles DMF and MMF also induces the same effects [36, 71, 72], which is the primary mechanism of their therapeutic action. Fumarate can also induce KEAP1 succination, but this primarily occurs under conditions of significantly elevated fumarate levels, such as FH deficiency, and is considered one of the mechanisms underlying its carcinogenic effects [73]. Under pathological concentrations of fumarate accumulation, multiple cysteine‐containing proteins are succinated, leading to functional abnormalities. Additionally, NRF2 plays a dual role in carcinogenesis, both inhibiting carcinogenesis initiation and promoting the progression of already transformed cells [74], making fumarate more likely to be described as a carcinogenic metabolite in FH‐deficient tumors. In adipocytes with FH knockdown, fumarate levels were observed to increase by more than 20‐fold, along with succination of KEAP1; however, in mature adipocytes under high‐glucose conditions, despite elevated fumarate levels (fourfold to fivefold), neither mass spectrometry analysis nor immunoprecipitation detected succination of KEAP1 [75]. Further studies revealed that both increased endogenous fumarate levels and DMF treatment elevated ROS levels in adipocytes, potentially due to GSH depletion, suggesting that KEAP1 thiol oxidation or succination depends on fumarate and oxidant levels [75].

### 3.5 Immunoregulation

Current preclinical studies and clinical trials indicate that fumarate and FAEs possess a relatively mild yet broad capacity to modulate immune responses. In this section, we summarize the effects of fumarate and FAEs on diverse immune cell populations to illustrate their role as a metabolic bridge linking cellular metabolism with both innate and adaptive immunity. These findings highlight the emerging importance of fumarate in immune regulation and suggest its therapeutic potential not only in autoimmune diseases but also in other immune‐related disorders, including cancer.

#### 3.5.1 Innate Immune Cells

The metabolic state of macrophages participates in and determines the polarization direction. Stimulated by LPS, glycolysis of macrophages is enhanced and oxidative phosphorylation is decreased, so that ATP can be produced quickly to meet the needs of inflammatory reaction. Fumarate level is increased in activated macrophages, where it regulates macrophage activation and cytokine expression. The accumulation of fumarate in LPS‐stimulated macrophages can be attributed to glutamine anaplerosis, which involves the conversion of glutamine to glutamate and α‐KG, as well as the aspartate–argininosuccinate shunt [10, 15]. Specifically, glutamine anaplerosis increases the levels of α‐KG in the TCA cycle, along with downstream intermediates such as fumarate and oxaloacetate. In the mitochondria, oxaloacetate is converted into aspartate by enzymatic catalysis, which then enters the cytoplasm. In the cytoplasm, aspartate is converted into argininosuccinate by ASS1 and further broken down to produce fumarate. By modifying GAPDH, fumarate inhibits glycolysis in activated macrophages and influences their polarization towards the M1 phenotype [28]. Sustained expression and activity of FH are essential for maintaining an appropriate cytokine response, preventing dysregulated cytokine production caused by excessive fumarate accumulation in LPS‐stimulated macrophages [15]. In LPS‐stimulated macrophages treated with DMF, growth differentiation factor‐15, an anti‐inflammatory factor, was significantly upregulated, which could be attributed to NRF2 activation [15, 76]. DMF was also found to suppress LPS‐induced expression of IL‐1β and IL‐6 [15]. However, DMF/MMF has been shown to reduce the release and expression of IL‐10 in LPS‐stimulated macrophages, thereby increasing tumor necrosis factor (TNF) levels [15, 77]. IL‐10 is a pleiotropic cytokine primarily known for its anti‐inflammatory effects, and the impact of fumarate on IL‐10 may be mediated through the succination of c‐Fos, a transcription factor subunit essential for IL‐10 induction [77]. The dual inhibitory effect of DMF on macrophage cytokines not only suppresses excessive inflammatory responses but also ensures the regulation of immune balance. Furthermore, the accumulation of mitochondrial fumarate can lead to the release of mtRNA. In macrophages, LPS‐induced fumarate accumulation may promote mtRNA release by increasing the mitochondrial membrane potential, thereby activating pathways such as RIG‐I and enhancing IFN‐β production [15]. While it is established that fumarate accumulation causes alterations in mitochondrial morphology and mtDNA release in renal epithelial cells [5], thereby driving innate immunity, direct evidence for this effect in macrophages is lacking. Moreover, the mechanisms by which fumarate alters mitochondrial morphology, as well as its potential to simultaneously trigger the release of both mtDNA and mtRNA in macrophages, warrant further investigation.

Monocytes and neutrophils are also key cellular components of the innate immune system. Monocytes belong to the mononuclear phagocyte system, and the mechanism underlying the therapeutic efficacy of DMF in RRMS involves the inhibition of proinflammatory microRNAs and inflammatory factors such as IL‐6 [78]. Chronic inflammation is a hallmark of both psoriasis and MS, with neutrophil infiltration playing a significant role in these conditions. Studies involving psoriasis patients and in vitro experiments have demonstrated that DMF could inhibit neutrophil activation [79, 80].

Like metabolic alterations in macrophages, the TCA cycle in dendritic cells (DCs) stimulated by LPS or proinflammatory signals is also rewired, and accumulation of fumarate is a major feature [81]. Previous studies showed that MMF treatment was able to markedly attenuate the ability of LPS‐stimulated DCs to secrete IL‐12p70 and IL‐10, and LPS‐induced activation of NF‐κB was also suppressed [82]. Recently, it has been demonstrated that DMF/MMF treatment impacts the maturation and function of DCs, mainly manifested as reduced expression of major histocompatibility complex class II and costimulatory molecules such as CD86 and CD80 [83, 84]. In psoriasis, DCs induce Th1 cell activation through the release of IL‐12, leading to the production of IFN‐γ and subsequent promotion of psoriatic inflammation [82, 85]. FAEs inhibit Th1 cell activation and IFN‐γ production by affecting cytokine secretion by DCs, which may be one of their mechanisms in psoriasis treatment. At the same time, inhibition of DC maturation and function by FAEs through suppression of NF‐κB and ERK1/2‐MSK1 signaling may affect the activation status and thus innate immunity, as DCs are important antigen‐presenting cells [84, 86].

NK cells participate in the regulation of innate and adaptive immunity by interacting with DCs as well as secreting cytokines such as IFN‐γ [87]. A 2‐year observational study revealed a significant increase in NK cell counts, reaching 85.9%, in MS patients treated with DMF [88]. The activation of NRF2 signaling is likely the primary causative factor, as it has been found that NRF2 signaling activation favors an increase in the number of CD56^bright^ NK cells, and facilitates remission of MS [89]. What is more, MMF has been reported to enhance NKp46 expression on the surface of CD56^+^ NK cells and Granzyme B release, which contributes to NK cell lysis of target tumor cells [90]. However, this effect was not observed with DMF, and only a high concentration of MMF (100 µM) exhibited efficacy in inducing this alteration in CD56^+^ NK cells. In addition, MMF (1 and 100 µM) or DMF (100 µM) effectively upregulated CCR10 expression in IL‐2‐activated NK cells, thereby enhancing NK cell lysis of target tumor cells [91]. This suggests that elevating local active metabolite MMF levels, but not DMF, may contribute to enhancing CD56^+^ NK cell tumor lethality; whereas DMF may play an auxiliary role by increasing the number of CD56^+^ NK cells.

#### 3.5.2 Adaptive Immune Cells

T cells are key drivers of MS pathogenesis, and accumulating evidence indicates that the therapeutic efficacy of DMF is largely mediated through modulation of T‐cell responses, particularly those of CD4^+^ T cells. DMF treatment reduces the proportions of circulating CD4^+^ and CD8^+^ memory T cells and limits the migration of CD4^+^ T cells into the central nervous system [92]. Furthermore, DMF has been reported to remodel the metabolic profiles of both CD4^+^ and CD8^+^ T cells while simultaneously reducing their antioxidant capacity, thereby promoting T‐cell apoptosis in patients with MS [93]. These findings highlight the close interplay between cellular metabolism and T‐cell function.

Similar to macrophages, glycolysis is essential for the activation and effector functions of lymphocytes [94]. Therefore, inhibition of glycolytic metabolism may represent a common mechanism by which fumarate and DMF/MMF regulate different lymphocyte subsets. For instance, by blocking glycolytic pathways, DMF/MMF can inhibit the differentiation and cytokine production of naive CD4^+^ T cells under Th1 and Th17 polarization conditions, while promoting the differentiation of Treg cells under Treg polarization conditions [28]. Beyond CD4^+^ T cells, fumarate can also directly impair CD8^+^ T‐cell function. In FH‐deficient tumors, fumarate accumulation suppresses the activation and cytotoxic activity of infiltrating CD8+ T cells by inducing succination of zeta‐chain‐associated protein kinase 70 (ZAP70), thereby disrupting T‐cell receptor (TCR) signaling [43]. Notably, CD8^+^ T cells also contribute to tissue damage in MS [95]. Consistent with this, inhibition of IL‐17‐producing CD8^+^ T cells is considered one of the mechanisms underlying the therapeutic effects of DMF in MS [96, 97]. Whether this immunomodulatory effect similarly involves fumarate‐mediated succination of ZAP70 and subsequent attenuation of TCR signaling remains to be determined.

Beyond T cells, B cells also appear to be important mediators of the therapeutic effects of DMF in MS. In experimental autoimmune encephalomyelitis, DMF suppresses the antigen‐presenting capacity of B cells, thereby limiting their ability to activate T cells [98]. In addition, fumarate and DMF can directly regulate B‐cell function through post‐translational protein modification. Succination of the tyrosine kinase LYN attenuates B‐cell receptor signaling, leading to impaired B‐cell activation and effector function [6]. These findings suggest that fumarate derivatives not only modulate T‐cell responses but also reshape the crosstalk between T and B cells, thereby dampening adaptive immune activation.

Collectively, current evidence suggests that fumarate and DMF/MMF exert broad immunometabolic effects across both innate and adaptive immune cells. By regulating cellular metabolism and inducing protein succination, fumarate and DMF/MMF modulate the activation and function of inherent immune cells and adaptive immune cells, thereby coordinating inflammatory responses and immune homeostasis. The identification of cell type‐specific succinated signaling proteins, such as ZAP70 and LYN, further suggests that protein succination represents a key molecular mechanism linking metabolic remodeling to immune regulation. A deeper understanding of these mechanisms may facilitate the development of next‐generation fumarate‐based therapies that achieve more precise immunomodulation with fewer adverse effects.

### 3.6 Inflammatory Response

Oxidative stress and the inflammatory response are closely linked, as oxidative stress can activate several inflammatory signaling pathways, such as NF‐κB and NLRP3. Meanwhile, the inflammatory response generates a large amount of ROS, further enhancing oxidative stress. Here, we discuss the effects of fumarate, MMF, and DMF on inflammatory response using NF‐κB and NLRP3 pathways as examples.

The NF‐κB pathway is widely recognized for its prototypical role in promoting inflammation, primarily due to its involvement in promoting the expression of proinflammatory cytokines, including TNF‐α, IL‐1β, and IL‐6. This pathway involves three critical protein families: the NF‐κB family, the IκB (inhibitor of κB) family, and the IKK (IκB kinase) family. The NF‐κB family comprises five transcription factors, including p65 (RelA), RelB, c‐Rel, p105/p50, and p100/p52, which are central to the pathway's function. The IκB family and IKK family are critical for regulating NF‐κB activity by inhibiting and activating the transcription factors, respectively. In unstimulated cells, the unphosphorylated IκB protein binds to RelA and p50, sequestering them in the cytoplasm. Upon stimulation, phosphorylation of IκB, mediated by the activated IKK complex, leads to its ubiquitination and subsequent degradation [99, 100], ultimately activating proinflammatory gene expression. DMF treatment disrupts the phosphorylation of the classical IκB protein family member IκBα at the S32/36 sites, leading to a significant reduction in RelA nuclear translocation [34]. In fact, the phosphorylation of IκBα is induced by the activated IKKβ subunit. DMF binds to the reactive cysteine residues within the kinase domain of IKKβ, particularly C179, inhibiting its kinase activity [34, 35]. Additionally, DMF can directly modify RelA, weakening its nuclear translocation and DNA‐binding activity [45]. DMF/MMF treatment has been shown to effectively inhibit the NF‐κB pathway and reduce levels of proinflammatory cytokines IL‐1β, TNF‐α, and IL‐6. This effect has been confirmed in disease models such as chronic cerebral hypoperfusion [101], thioacetamide‐ or sepsis‐induced liver injury [102, 103], and septic acute kidney injury [104].

The NLRP3 inflammasome is a protein complex that serves as a critical mediator of inflammation and plays a crucial role in immune‐ and inflammation‐related diseases. The primary components of the NLRP3 inflammasome include the sensor NLRP3, the adaptor ASC (apoptosis‐associated speck‐like protein containing a caspase recruitment domain), and the effector Caspase‐1. These components are responsible for sensing danger signals and recruiting downstream molecules, linking the other two molecules, and promoting the maturation of cytokines IL‐1β and IL‐18, as well as the processing of GSDMD [105, 106]. Studies have shown that DMF can reduce the levels of key elements of the NLRP3 inflammasome in microglial cells stimulated by LPS or ATP, which is associated with the inhibition of NF‐κB signaling [107]. This leads to the suppression of NLRP3 inflammasome assembly and activation, as well as subsequent pyroptosis and inflammation. Furthermore, the effect of DMF on NLRP3 and Caspase‐1 expression can be abolished by NRF2 inhibitors, indicating that DMF also influences NLRP3 inflammasome activation through the NRF2 pathway. This has been confirmed in a rat model of bilateral cavernous nerve injury [108]. Fumarate and FAEs can also directly disrupt the processing of GSDMD by Caspase‐1 through GSDMD succination, impairing pore formation in the membrane [29].

### 3.7 Cell Death

The death of damaged or discarded cells is essential for maintaining homeostasis, while impaired or excessive activation of cell death processes may promote the development of diseases. Recent findings have indicated that fumarate and FAEs could regulate various modes of programmed cell death.

Apoptosis is a form of programmed cell death mediated by Caspase‐3/7 and involving multiple molecules [109]. DMF can induce apoptosis in specific cells, and its mechanism is related to inhibiting NF‐κB activity. p53, one of the classic tumor suppressor genes, can activate the intrinsic apoptotic pathway, while the NF‐κB signaling pathway suppresses apoptosis by influencing p53 [110]. DMF treatment is beneficial for restricting the growth and metastasis of cutaneous T‐cell lymphoma, and the involved mechanism is the inhibition of NF‐κB activation [111, 112, 113]. The activation of NF‐κB is also a crucial factor in the progression of triple‐negative breast cancer [114]. After treatment with DMF, the expression of apoptosis inhibitors Survivin, X‐linked inhibitor of apoptosis (XIAP), B‐cell lymphoma extra‐large (Bcl‐xL), and Bcl‐2 in cancer cells is suppressed, further supporting the proapoptotic effect of DMF [114]. Interestingly, other studies have identified the apoptotic inhibition properties of FAEs, which have been confirmed in models of pulmonary IRI as well as in polymorphonuclear neutrophils [115, 116]. Although FAEs treatment can reduce IR‐induced IκB‐α expression and NF‐κB p65 nuclear translocation, it exhibits effects that inhibit cleaved Caspase‐3 expression and lower the Bax/Bcl‐2 ratio. This may be related to inhibiting the interaction between GAPDH and Siah1, thereby preventing their nuclear translocation [115]. Similarly, DMF can also lower the Bax/Bcl‐2 ratio in high‐glucose‐treated retinal pigment epithelial (RPE) cells, thereby suppressing apoptosis [117]. In blue‐light‐induced apoptosis of RPE cells, DMF primarily confers resistance to apoptosis by activating the NRF2 signaling pathway [118]. Collectively, the influence of fumarate and FAEs on apoptosis is determined by various signaling pathways, with cell type and state potentially being key factors.

Pyroptosis is a form of programmed cell death executed by proteins of the gasdermin family, which induces a robust inflammatory response [119]. GSDMD is the first identified executor of pyroptosis, containing cysteine residues that can be modified by fumarate [29]. The application of FHIN1 resulted in reduced formation of GSDMD‐N in LPS‐activated macrophages, thereby inhibiting cell death [29]. GSDMD‐N is a fragment released after GSDMD cleavage, which oligomerizes to form pores in the plasma membrane, facilitating the release of IL‐1β and IL‐18. Exogenous fumarate, namely, DMF, exhibits similar effects and prevents the occurrence of experimental autoimmune encephalomyelitis in animal models [29]. In short, succinated GSDMD loses the ability to interact with caspase, hindering its processing and oligomerization, thereby limiting pore formation, cytokine release, and cell death. In macrophages, DMF can also inhibit the production of Type I IFN induced by LPS, as well as the subsequent expression of Caspase‐11, which processes GSDMD to mediate pyroptosis [120]. Macrophage infiltration, polarization to the M1 phenotype, and pyroptosis are important features of acute kidney injury in mouse models of crush syndrome [121]. Administration of DMF alleviates kidney tissue damage by suppressing macrophage polarization and pyroptosis. Beyond macrophages, DMF can also inhibit GSDMD‐induced pyroptosis in intestinal resident intraepithelial lymphocytes, thereby reducing intestinal inflammation [122]. In addition to GSDMD, fumarates also affect the processing of other members of the gasdermin family, such as GSDME and GSDMB [29, 123].

Necroptosis, another form of programmed cell death, is mediated by receptor‐interacting protein kinases 1 and 3 (RIPK1/3) and mixed lineage kinase domain‐like protein (MLKL). Studies have found that DMF can inhibit necroptosis in macrophages induced by a combination of TNF‐α, Smac mimetic LCL‐161, and the pan‐caspase inhibitor IDN‐6556 (TSI), or by LPS and IDN‐6556 combined [124]. Further research suggests that DMF's electrophilic nature is responsible for its inhibitory effect on necroptosis by blocking TSI‐induced reverse electron transport and phosphorylation of RIPK1, RIPK3, and MLKL, thereby preventing the activation of the RIPK1–RIPK3–MLKL axis [124]. Similarly, using FHIN1 to inhibit FH activity significantly suppresses TSI‐induced necroptosis. Another study indicated that fumarate accumulation, resulting from reduced FH activity, can suppress necroptosis in nasopharyngeal carcinoma cell HK1 and nasopharyngeal epithelial cells NP460hTERT by increasing the methylation levels of the *RIPK3* promoter, thus inhibiting its expression [125]. This effect is achieved by inhibiting the enzymatic activity of TET family of DNA hydroxylases, as fumarate acts as an α‐KG antagonist.

Ferroptosis is also a form of programmed cell death, which is manifested by the accumulation of free divalent iron in the cytoplasm, imbalance of the antioxidant system, and accumulation of lethal lipid peroxides [126]. The main pathways of ferroptosis include imbalance of the oxidative system caused by the cystine/GSH/GPX4 (glutathione peroxidase‐4) pathway, abnormal iron metabolism, and abnormal lipid peroxidation metabolism. Many previous studies have reviewed this in detail, which will not be repeated here. At present, there are some contradictions about the relationship between fumarate and ferroptosis. Some studies have shown that the loss of FH function increases the resistance of cells to cysteine deprivation‐induced ferroptosis [127]. A recent study also showed that theabrownins can reduce renal cell apoptosis and ferroptosis caused by burns by increasing the content of fumarate in the kidney [128]. However, in tumor‐related studies, when FH is inactive, the accumulated fumarate can easily modify the Cys93 site of GPX4, and this modification will inhibit the activity of GPX4, thereby inducing tumor cell ferroptosis [44]. In addition, a review explored the ferroptosis mechanism of FH‐related tumors [129]. Mutations in the FH gene can alter cell metabolism and increase the risk of tumor development, especially in the kidneys. Since most tumor cells require a higher number of ferrous ions than normal cells, they are more susceptible to ferroptosis. In addition, knocking down ASL to reduce intracellular fumarate levels can alleviate Erastin‐induced ferroptosis [130]. The above studies have demonstrated that there is a close connection between abnormal fumarate levels and ferroptosis, but the direct relationship has not been clearly elucidated. The different conclusions between these studies may come from different model construction methods. For example, fumarate may play different roles in ferroptosis models induced by cysteine deprivation or ferroptosis inducers. In addition, the role of fumarate may also be different due to differences between tumor cells and other types of cells.

In addition to fumarate itself, DMF has also been found to have a bidirectional regulation of ferroptosis. Studies have found that the survival rate of most diffuse large B‐cell lymphoma (DLBCL) cell lines was significantly reduced after DMF treatment. DMF can effectively reduce GSH levels and induce lipid peroxidation, leading to ferroptosis. Based on this result, researchers have developed a nanosystem containing DMF and other drugs. DMF can directly reduce GSH levels in tumor cells by inhibiting system Xc‐, thereby promoting lipid peroxidation and ferroptosis. These effects have been verified in the germinal center B‐cell‐like‐DLBCL mouse model [131]. In addition, DMF, as an Nrf2 activator, plays a protective role in nontumor diseases by inhibiting ferroptosis. Its mechanism is closely related to regulating specific pathways and molecules and maintaining cell homeostasis. In the liver IR model, ferroptosis can be inhibited by activating the NRF2/SLC7A11/HO‐1 (heme oxygenase‐1) axis [132]. In traumatic brain injury, DMF‐mediated NRF2 activation can alleviate neurological dysfunction in mice, partly due to the inhibition of ferroptosis. NRF2 counteracts ferroptosis by regulating xCT–GPX4, FTH–FTL (involving free iron levels), and FSP1 (involving oxidative status) [133]. In addition, in diseases such as acute kidney injury, alcoholic liver disease, and sickle cell disease, DMF can also inhibit ferroptosis by targeting NRF2, regulating ROS, GSH, and lipid peroxidation [134, 135, 136, 137, 138]. In addition to targeting NRF2, DMF can also reduce sepsis‐induced lung ferroptosis, inflammation, and oxidative damage by inhibiting STING activation and preventing GPX4 degradation [139]. Based on the current research, we found that DMF can promote ferroptosis in tumors and inhibit ferroptosis in IRI, neurodegenerative diseases, respiratory distress syndrome, and other diseases. We believe that its tumor cell ferroptosis‐promoting effect is because tumor cells have active metabolism, high iron accumulation, and possible defects in antioxidant mechanisms. Normal cells benefit from the activation of NRF2 by FAEs and acquire a certain resistance to ferroptosis. In general, DMF can play a protective role in the body by resisting or promoting ferroptosis in a variety of diseases.

## 4 Therapeutic Potential of Fumarate and FAEs Across Diseases

We introduce the metabolism of fumarate and FAEs represented by MMF and DMF, the differences in their inherent properties, especially electrophilicity, and the effects on physiological and pathological processes. Compared with fumarate itself, FAEs have a wider range of targeting capabilities as drugs, and they can be gradually hydrolyzed into fumarate in the body and metabolized, so that they can eventually be metabolized and cleared by cells, reducing toxic accumulation. Table 3 provides a comprehensive summary of the functions and related mechanisms of FAEs in diseases. Given the promising therapeutic potential of FAEs, we present the central pathways in Figure 5. Since the application of FAEs in the treatment of MS and psoriasis has been systematically described in many reviews, we will not repeat it here. In addition, fumarate exists in some drugs as excipients or base forms, such as tenofovir alafenamide fumarate, which can improve the stability, water solubility, and bioavailability of drugs, and the related content is beyond the scope of this review.

**TABLE 3:** Summary of FAEs in disease and the related signaling pathways.

Disease	Mechanism	Clinical significance	References
Neurodegenerative disease	Energy metabolism; Activate NRF2 and hydroxycarboxylic acid receptor 2 (HCAR2) signaling; Inhibit NF‐κB pathway and the activation of NLRP3 inflammasome	Neuroprotective effects; Alleviate cognitive and motor impairments; First‐line drug for (RR)MS	[140, 141]
Cardiovascular disease	Energy metabolism; Activate NRF2 pathway; Inhibit the activation of early growth response‐1 (Egr‐1)	Improve cardiac function; Attenuate myocardial injury	[142]
Cerebrovascular disease	Energy metabolism; Activate NRF2 pathway; Inhibit immune cell infiltration	Alleviate cerebral infarction and edema; Potential therapeutic agents	[101, 143]
Hepatic disease	Energy metabolism; Activate NRF2 pathway; Inhibit the NF‐κB pathway and the activation of NLRP3 inflammasome	Prevent the progression of liver fibrosis; Inhibit the progression of fatty liver	[103, 144, 145]
Respiratory disease	Prevents the processing of GSDMD/GSDME; Activate NRF2 pathway	Reduce lung inflammation and relieve pulmonary fibrosis	[138, 146, 147]
Eye disease	Inhibit the NF‐κB pathway and the activation of NLRP3 inflammasome; Activate NRF2 and HCAR2 signaling	Neuroprotective effects; Prevent pathological angiogenesis; Potential therapeutic agents	[148, 149]
Cancers	Induce cell death; Inhibit NF‐κB pathway; Inhibit NRF2 pathway at high concentrations	Potential therapeutic agents	[45, 150]

*Abbreviations*: HCAR2, hydroxycarboxylic acid receptor 2; NF‐κB, nuclear factor κ‐light‐chain‐enhancer of activated B cells; NLRP3, NOD‐, LRR‐, and pyrin domain‐containing protein 3; NRF2, nuclear factor erythroid‐2‐related factor 2.

**FIGURE 5:**
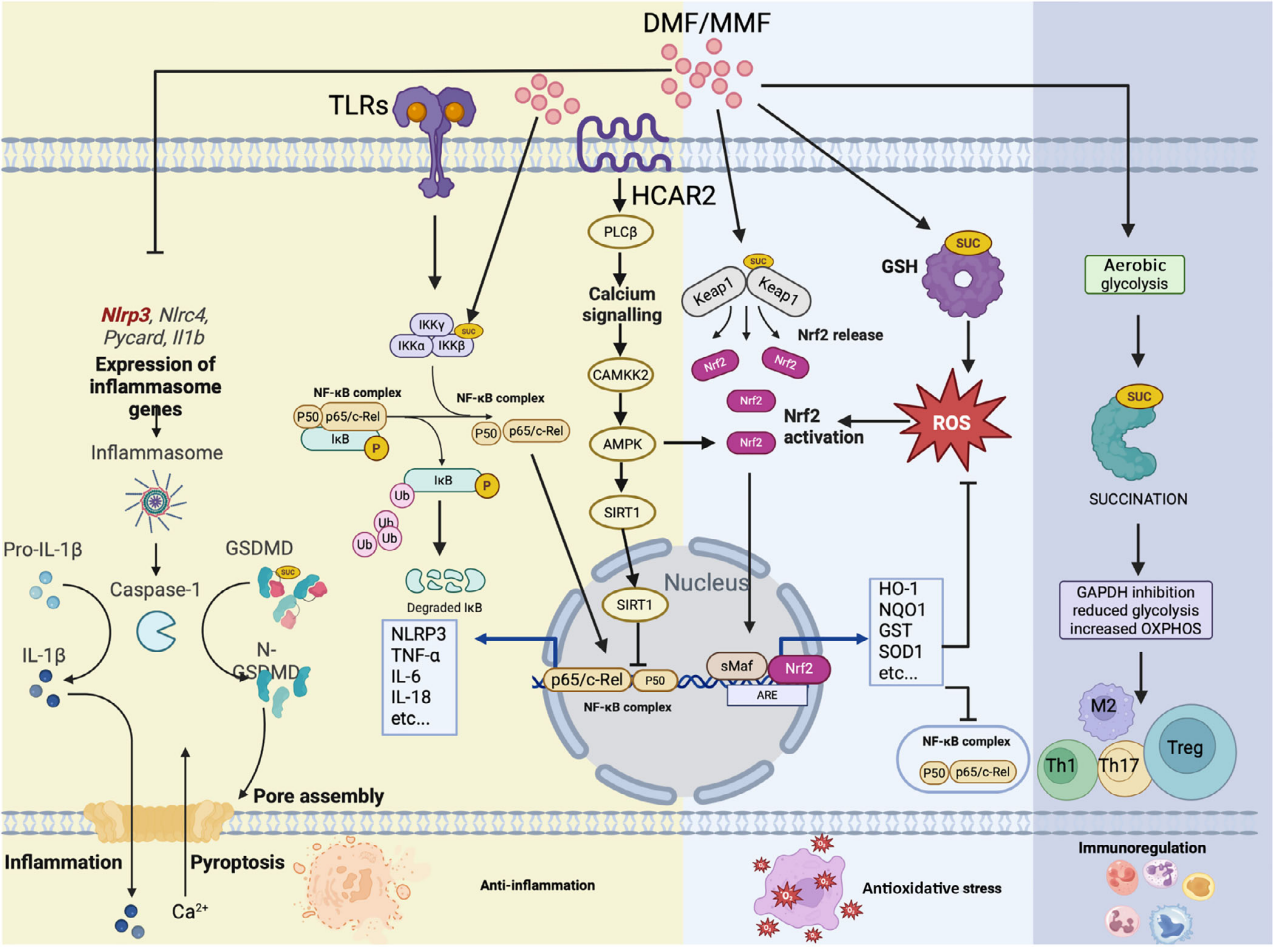
The main signaling pathways involved in the therapeutic effects of DMF/MMF in disease treatment. DMF/MMF modifies IKKβ to inhibit the NF‐κB signaling pathway, thereby reducing the expression of inflammatory factors while also suppressing NLRP3 expression. By directly modifying GSDMD, DMF/MMF enhances its ability to inhibit pyroptosis and exert anti‐inflammatory effects. Modification of KEAP1 activates the NRF2 signaling pathway, promoting the expression of a series of genes that contribute to antioxidant, anti‐inflammatory, and cytoprotective functions. The succination of GAPDH inhibits aerobic glycolysis in activated immune cells, playing a role in immune regulation. TLRs, Toll‑like receptors; HO‐1, heme oxygenase‐1; NQO1, NAD(P)H:quinone oxidoreductase 1; GST, glutathione‐S‐transferase; SOD1, superoxide dismutase 1; AMPK, AMP‐activated protein kinase; SIRT1, silent information regulator sirtuin 1.

### 4.1 Oxidative Stress‐Related Diseases

Oxidative stress is a component of many diseases. The cellular effects caused by different degrees of oxidative stress are closely related to cardiovascular and cerebrovascular diseases, neurodegenerative diseases, inflammation, and tumors. To resist oxidative damage, organisms have evolved defense mechanisms that mainly rely on antioxidant enzymes, substrate supply, and damage repair. These defense mechanisms are enhanced in the presence of oxidants and electrophiles, thereby enhancing the ability to detoxify oxidants, electrophiles, and repair damage [151]. The NRF2 signaling pathway is currently considered to be one of the most important antioxidant mechanisms. FAEs, as derivatives of the electrophilic reagent fumarate, especially NRF2 activators, have great potential in enhancing the body's defense mechanisms and treating oxidative stress‐related diseases.

In many diseases, oxidative stress is often secondary to other factors, such as inflammation and IR. Oxidative stress affects a variety of biological processes by interfering with various signaling pathways and impairing organelle function, accelerating pathological progression. Many studies have revealed that FAEs have a significant effect in reducing oxidative stress and inflammatory responses in IRI. In an acute myocardial infarction model, DMF improved cardiac function by activating the NRF2 signaling pathway. This was reflected by a reduction in both end‐diastolic and end‐systolic left ventricular diameters, as well as lower serum levels of cardiac injury markers CK‐MB and cTnT [152]. Interestingly, DMF's differential regulation of cellular metabolism also aids in reducing ischemic injury to the heart. For example, DMF enhances oxidative phosphorylation in macrophages while impairing it in fibroblasts, resulting in anti‐inflammatory and tissue‐repair effects during myocardial infarction [153]. The NRF2‐activation effects mediated by DMF have also been reported in IRI of other organs such as the liver, intestine, kidney, and skin [145, 154, 155, 156].

Unlike IRI, where oxidative stress is an important factor in disease progression, it can also be the main cause of certain diseases, such as atherosclerosis and toxicity caused by radiation and paraquat [151]. Taking atherosclerosis as an example, oxidative stress can lead to the conversion of low‐density lipoprotein (LDL) cholesterol into pathogenic oxidized LDL, which can promote the progression of atherosclerosis by initiating and promoting inflammatory responses and leukocyte recruitment [157]. Several studies have found that DMF has the effect of improving the ratio of high‐density lipoprotein (HDL)/LDL and HDL/total cholesterol in the treatment of MS and psoriasis [158, 159]. In addition, DMF also has the effect of improving endothelial cell function, which is closely related to the activation of NRF2 signaling [160]. In summary, FAEs are expected to become an important therapeutic strategy for oxidative stress‐related diseases.

### 4.2 Ischemic Disease

Insufficient blood supply to an organ or tissue often signifies a lack of oxygen and nutrients, leading to metabolic disorders and subsequent dysfunction. During ischemia, glycogen is broken down into lactate and ATP, and the elevated lactate levels inhibit ATP production, impair ion transport proteins, and induce the release of inflammatory factors, thus exacerbating tissue damage [161]. Upon reperfusion, a large amount of oxygen free radicals is generated, causing further damage to tissues and cells [162]. For instance, in ischemic stroke, ischemia and reperfusion‐induced capillary dysfunction can lead to the breakdown of the blood‐brain barrier, resulting in brain edema and causing more significant damage to brain tissue [163, 164]. In addition to the brain, organs such as the heart, kidney, liver, and retina are also highly susceptible to ischemic injury due to their high metabolic demands and specialized physiological functions.

From a metabolic perspective, besides lactate, TCA cycle intermediates such as fumarate and succinate levels also change during tissue ischemia. Studies have found that succinate accumulates during tissue ischemia [165]. Chouchani et al. suggested that the accumulation of succinate during cardiac ischemia is primarily due to the reverse activity of SDH, where the reversal from fumarate to succinate represents the optimal mechanism for maintaining proton pumping and ATP production when fumarate, aspartate, and malate are available [165]. The activation of the malate–aspartate shuttle and the purine nucleotide cycle pathways during ischemia increases fumarate production, facilitating this optimal mechanism [165]. However, Zhang et al. argued that the primary mechanism for the increased succinate production during cardiac ischemia is not SDH reversal but rather classic TCA cycle activity [166]. The source of succinate during tissue ischemia remains contentious [167], but it is undeniable that the reversal from fumarate to succinate occurs and contributes to mitigating ischemic damage. During reperfusion, SDH's forward activity is restored, and the accumulated succinate is rapidly oxidized to fumarate to maintain the reduced Q pool, establishing the conditions for a strong proton motive force, which drives reverse electron transfer at Complex I, producing ROS that induce IRI [165]. One study used LC–MS/MS analysis on the plasma of pigs subjected to transient coronary occlusion (40 min), showing that plasma samples from the great cardiac vein had significantly elevated concentrations of succinate, fumarate, malate, citrate, and lactate after IR, peaking at approximately 5 min of reperfusion [168]. Further research revealed that compared with the control group, the use of SDH inhibitor malonate, remote ischemic preconditioning, or combined therapy as cardioprotective measures did not reduce the increase in succinate during reperfusion but significantly lowered fumarate and malate levels [168]. Compared with the remote ischemic preconditioning group, fumarate and malate levels were even lower in the malonate or combined therapy groups [168], indicating that the source of fumarate and its downstream product malate during reperfusion is primarily succinate oxidation.

One of the key points of IRI treatment is to alleviate the injury, and the key is antioxidation, anti‐inflammation, and immunomodulation. It is well known that fumarate possesses antioxidant and immunomodulatory properties. However, a recent study presents that mitochondrial‐derived fumarate may promote IR‐induced tubular injury [169], which appears contradictory. Similar to myocardial IRI, they found that fumarate levels increase during renal IR and are primarily localized in the cytoplasm [169]. Enhanced glycolysis and mitochondrial dysfunction in renal tubules are also important features during renal IRI. They suggested that fumarate accumulation is associated with abnormalities in FH, as both FH transcription and protein levels in kidney decrease after IR. Another study indicated that after renal IRI, both mitochondrial and tissue levels showed reduced FH activity, while FH activity was enhanced in plasma and urine [170]. One hypothesis is that IRI causes damage to the plasma membrane and mitochondria, leading to subsequent release of FH into the extracellular space [170]. This may explain the observed decrease in renal tissue FH levels reported in these two studies. At the onset of reperfusion in the kidneys, using the SDH inhibitor 3‐nitropropionic acid can inhibit the increase in fumarate levels and reduce tissue damage [169]. Additionally, in HK‐2 cells treated with MMF, the levels of inflammatory factors such as IL‐1β and TNF‐α were found to be elevated. The authors believe that the main mechanism by which the SDH inhibitor exerts its protective effect is through the inhibition of the increase in fumarate levels [169]. This seems reasonable; however, it was reported that succinate would accumulate during renal ischemia, and inhibiting this process was helpful to protect mitochondrial dysfunction and alleviate renal injury during reperfusion [171, 172]. In other words, it is important to note that the use of SDH inhibitors can also inhibit SDH‐mediated succinate oxidation, thereby reduce the production of ROS and contributing to tissue protection [165, 171]. It is worth mentioning that another study found that DMF has a protective effect in rat models of renal IRI [155]. The expression levels and activity of FH, along with the changes and roles of succinate and fumarate levels during kidney and other tissue IR, merit further exploration.

Regarding FAEs, current research predominantly supports their protective role in tissue IRI. Studies have found that MMF can significantly reduce cortical neuronal death and proinflammatory cytokine release caused by oxygen‐glucose deprivation in vitro. In animal models, DMF/MMF significantly reduced the size of infarcts or cerebral edema and alleviated neurological deficits caused by middle cerebral artery occlusion in rats and mice [72, 173]. Mechanistically, the protective effects of FAEs in experimental stroke models are mainly achieved through the activation of the NRF2 pathway, inhibition of immune cell infiltration, and modulation of inflammation levels [72, 173]. In an acute myocardial infarction model established by ligating the left anterior descending coronary artery, DMF improved cardiac function by activating the NRF2/HO‐1 signaling pathway in the heart. This was reflected by a reduction in both end‐diastolic and end‐systolic left ventricular diameters, as well as lower serum levels of cardiac injury markers CK‐MB and cTnT [152]. Additionally, DMF inhibited ischemia‐induced activation of the transcription factor early growth response‐1 in acute myocardial infarction cell models. Early growth response‐1 activation triggers signaling cascades that lead to the expression of chemokines, adhesion molecules, coagulation factors, and factors related to endothelial permeability, which contribute to vascular dysfunction during reperfusion [142, 174]. Interestingly, DMF's differential regulation of cellular metabolism also aids in reducing ischemic injury to the heart. For example, DMF enhances OXPHOS in macrophages while impairing OXPHOS in fibroblasts, resulting in anti‐inflammatory and tissue‐repair effects during myocardial infarction [153]. The NRF2‐activation effects mediated by DMF have also been reported in IRI of other organs such as the liver, intestine, kidney, and skin [145, 154, 155, 156], although these will not be discussed in detail here.

### 4.3 Nervous System Diseases

Nervous tissue is highly susceptible to damage caused by factors such as trauma, ischemia, hypoxia, inflammation, and immune dysfunction. Recent findings suggest that fumarate significantly protects both central and peripheral nervous tissues, primarily through its antioxidant, anti‐inflammatory, and immunomodulatory actions.

Multiple studies have demonstrated that fumarate and FAEs could alleviate the progression of various neurodegenerative diseases. Current research on fumarate in the context of neurodegenerative diseases primarily focuses on Alzheimer's disease (AD), Parkinson's disease (PD), and MS. The administration of streptozotocin via ventricles is a commonly used method to establish the AD model, which induces oxidative stress and neuroinflammation, leading to hippocampal neurodegeneration, cholinergic deficits, and brain energy metabolism disorders [175]. Treatment with DMF significantly mitigates hippocampal inflammation and neuronal damage induced by streptozotocin, thereby improving spatial cognitive ability [176, 177]. In other AD models, DMF has also exhibited considerable neuroprotective effects [178, 179]. The efficacy of DMF is attributed to the induction of NRF2 and its target genes, which help to inhibit lipid peroxidation, mitochondrial dysfunction, cell death, inflammation, and amyloid‐β deposition [178, 180]. In animal models of PD induced by 6‐hydroxydopamine and 1‐methyl‐4‐phenyl‐1,2,3,6‐tetrahydropyridine, as well as in transgenic mice, DMF exerts similar therapeutic effects through the NRF2 pathway [181, 182, 183]. The most crucial role of DMF in PD animal models is its neuroprotective effect on the substantia nigra, helping to maintain dopamine levels and its metabolites in the striatum [184].

DMF also exhibits protective effects in non‐neurodegenerative diseases of the nervous system. In ischemic stroke, neuronal damage primarily results from ischemia, hypoxia, and subsequent oxidative stress due to vascular occlusion [173]. Research has shown that DMF protects cells in the infarcted area from oxidative stress damage through the NRF2 pathway. Additionally, DMF significantly suppresses the activation of microglia in the penumbra and surrounding brain regions and dramatically reduces the infiltration of potentially destructive immune cells into the infarcted area, providing multidimensional protection to brain tissue [173]. It is noteworthy that DMF could affect major glial cells of the central nervous system, including oligodendrocytes, astrocytes, and microglia. Reports indicate that treating oligodendrocytes with DMF can influence changes in their TCA cycle intermediates, GSH, and lipids, thereby exerting an antioxidant effect [185]. For microglia, fumarate can convert them from a proinflammatory phenotype to an anti‐inflammatory phenotype, inhibiting the expression of various neuroinflammatory mediators [141, 186]. In astrocytes, DMF significantly reduces the secretion of IL‐6, CXCL10, and CCL2, as well as ROS production, while also suppressing the expression of proinflammatory miR‐146a and ‐145 [187].

Fumarate and FAEs also show therapeutic potential in peripheral nerve injury and can promote nerve regeneration. In a rat model of bilateral cavernous nerve injury, DMF treatment improved the morphology of the cavernous nerves, increased the number of nerve fibers, and enhanced erectile function in the rats [108]. In addition to its antioxidant effects, fumarate also demonstrated the ability to inhibit NLRP3 inflammasome activation and pyroptosis in this model. After sciatic nerve compression in mice, administration of DMF not only reduced inflammation levels but also promoted axonal regeneration [188, 189]. Szepanowski et al. suggested that the regenerative potential of DMF may be mediated by the upregulation of HO‐1 in Schwann cells and neurons [189].

In addition to protecting the central and peripheral nervous systems from injury, fumarate also appears to alleviate neuropathic pain. Studies have shown that DMF helps reduce nociceptive hypersensitivity induced by peripheral nerve injury in mice, which is associated with its antioxidant stress activity [190]. By activating the NRF2 pathway, DMF increases superoxide dismutase activity following nerve injury, reduces the levels of cytokines and chemokines (such as IL‐1β and CCL2), and mitigates mitochondrial dysfunction. In a mouse model of neuropathic pain induced by chronic constriction of the sciatic nerve, oral DMF also demonstrated analgesic effects, reducing levels of NLRP3 in the dorsal root ganglia and amygdala, while increasing or restoring the levels of NRF2, HO‐1, and superoxide dismutase‐1 [191]. Interestingly, DMF also helps alleviate emotional disturbances caused by neuropathic pain. In a mouse model of neuropathic pain, the upregulation of hydroxycarboxylic acid receptor 2 (HCAR2) was observed in the sciatic nerve and dorsal root ganglia [192]. Furthermore, long‐term use of DMF was shown to reduce the firing activity of ON cells in the rostral ventromedial medulla, which are typically associated with enhanced pain perception [192].

### 4.4 Ocular Diseases

Age‐related macular degeneration (AMD) is one of the leading causes of vision loss or blindness worldwide, and RPE cell dysfunction plays a central role in the pathology [193]. Targeting the NRF2 pathway helps prevent oxidative stress‐induced RPE damage, and FAEs, as NRF2 activators, have significant potential in treating AMD [194]. TNF‐α treatment induces mitochondrial dysfunction in RPE cells, and DMF pretreatment significantly suppresses TNF‐α‐induced release of proinflammatory cytokines such as IL‐6 and IL‐8, complement activation, and restores mitochondrial health, thus normalizing cellular metabolic function [195]. RPE cells affected by TNF‐α exhibit increased aerobic respiration and decreased glycolysis [195], a metabolic shift that could affect the nutrient and oxygen supply to adjacent retinal cells. Excessive light exposure can damage photoreceptors, leading to retinal injury [196]. In fact, photoreceptor degeneration is considered a common hallmark of various retinal degenerative diseases, such as dry AMD [197]. Jiang et al. found that prior administration of MMF could adequately protect the retinal structure and function in a light‐induced retinal degeneration mouse model [149]. After exposure to intense light, the expression of HCAR2 in microglial cells was upregulated, which may represent an intrinsic protective mechanism. As an HCAR2 receptor agonist, MMF converts microglia from a proinflammatory phenotype to an anti‐inflammatory phenotype, thereby reducing neuroinflammation and exerting a protective effect on the retina [141, 149].

Glaucoma is the leading cause of vision loss and is characterized by retinal ganglion cell (RGC) death, optic nerve damage, and visual field impairment. Currently, lowering intraocular pressure is the primary means of treating glaucoma; however, the role of oxidative stress in the pathogenesis of glaucoma cannot be overlooked. Several studies have reported elevated oxidative stress markers in the aqueous humor, trabecular meshwork, and serum of glaucoma patients [198, 199]. Interestingly, ROS is also a potential factor contributing to increased intraocular pressure, as it can induce apoptosis in trabecular meshwork cells, disrupt the extracellular matrix of these cells, and promote trabecular meshwork cell fusion, thereby impeding aqueous outflow [200]. It has been established that ROS can promote apoptosis of RGCs and lead to dysfunction of the glial cells in the posterior segment, contributing to the pathogenesis of glaucoma [201]. The relationship between oxidative stress and glaucoma is complex, and we will not elaborate further due to space limitations. Retinal IR is a commonly used model for exploring the pathogenesis of glaucoma. Research has reported that DMF/MMF can activate the NRF2 pathway during retinal IR, thereby reducing Müller cell gliosis and neuronal cell loss in the ganglion cell layer, improving retinal function [202]. The optic nerve crush model is commonly used to investigate neuroprotective and regenerative mechanisms. Research has shown that DMF can promote the survival of RGCs following optic nerve crush via the NRF2/HO‐1 pathway [203], providing a basis for treating glaucoma and other ocular diseases.

Diabetic retinopathy (DR) is a major ocular complication of diabetes and imposes a significant global health burden. DR has long been regarded as a microvascular complication; however, numerous studies have found that diabetic optic nerve degeneration is an early event in the pathogenesis [204]. Activation of the NRF2 pathway by fumarate and FAEs also contributes to the treatment of DR, as a reduction or loss of NRF2 activity has been observed in this disease [205]. Compared with normal retinas, the expression of HO‐1 is increased in the total thickness of the retinas of diabetic rats, which may serve as a self‐defensive mechanism. In a streptozotocin‐induced diabetic rat model, administration of DMF can reduce retinal iNOS and COX‐2 levels, and significantly increase HO‐1 expression, thereby combating the inflammatory response and oxidative stress associated with DR [206]. When exposed to high glucose, the phosphorylation level of p38 MAPK in RPE cells will increase significantly, and DMF can partially counteract this effect, promote cell survival, and reduce inflammation [117]. Vascular endothelial growth factor levels are also downregulated after DMF treatment [117], indicating that DMF may help prevent angiogenesis in DR and slow disease progression.

Excessive inflammatory response is a major characteristic of keratitis and uveitis. In a study using mice infected with herpes simplex virus 1 (KOS strain), severe herpes stromal keratitis developed in most mice by Day 14 postinfection. However, after intraperitoneal injection of DMF, viral keratitis was significantly improved, and this effect was associated with a reduction in T cells and monocyte infiltration in the cornea [207]. In a fungal keratitis mouse model, DMF significantly reduced corneal fungal load and alleviated corneal damage by inhibiting the accumulation of inflammatory cells and downregulating cytokine expression. Specifically, DMF markedly decreased the fungal‐induced high expression of NLRP3, GSDMD‐N, cleaved‐Caspase‐1, IL‐1β, and IL‐18 [208]. In experimental autoimmune uveitis, DMF alleviated uveitis symptoms by restoring the balance between effector T cells and regulatory T cells, reducing effector T cell infiltration by inhibiting PIM1 and CXCR4 expression [209].

### 4.5 Cancers

Although fumarate is described as a potential carcinogenic metabolite, this mainly occurs in the context of FH deficiency. In preclinical studies, the anticancer potential of FAEs is gradually being revealed.

#### 4.5.1 Colorectal Cancer

Colorectal cancer (CRC) is one of the leading causes of cancer‐related mortality worldwide, characterized by the late manifestation of symptoms during disease progression. Emerging evidence suggests that DMF exhibits antitumor activity in CRC. Research has shown that treatment with DMF significantly reduces the viability of various CRC cell lines, such as T84 and CT26 [210, 211]. DMF enhances Caspase‐3/7 activity in a dose‐dependent manner in T84 cells, leading to apoptosis. In CT26 cells, high concentrations of DMF trigger necroptosis by depleting GSH and increasing ROS levels. In a mouse model of CRC, DMF (20 mg/kg) has been shown to prevent chemically induced tumor formation and inhibit the growth of established tumors [150]. However, further studies have demonstrated that MMF at the same concentration has minimal impact on cell viability and does not induce cell death [211]. Similarly, low concentrations of DMF do not exert cytotoxic effects on CRC cells. Saidu and colleagues have highlighted that the NRF2 pathway is critical for the cytotoxicity of high‐dose DMF, achieved through inhibiting NRF2 stabilizer DJ‐1 expression [150]. The regulatory effect of DMF on the NRF2/DJ‐1 axis was also confirmed in the ovarian cancer cell line OVCAR3 [150].

#### 4.5.2 Melanoma

Melanoma is a malignant tumor derived from melanocytes, which mostly occurs in the skin. DMF has demonstrated the ability to impair melanoma growth and metastasis in mouse models, mainly due to its antiproliferative and proapoptotic effects [212]. In melanoma, activation of NF‐κB promotes cancer cell survival and migration. DMF has been found to inhibit nuclear translocation of NF‐κB in melanoma cells, thereby inhibiting this pathway [213, 214]. Inhibition of NF‐κB resulted in reduced expression of downstream matrix metalloproteinases, very late antigens, survivin, and Bcl‐xL, among others, which is the reason why DMF inhibits tumors from many aspects. In addition, the inhibitory effect of DMF on NF‐κB pathway may also help to enhance the efficacy of other chemotherapeutic agents and reduce the development of drug resistance [215]. However, no changes in IκB phosphorylation levels in DMF‐treated cells were observed [213], suggesting that DMF may inhibit NF‐κB by other means, such as direct covalent modification [45].

#### 4.5.3 Hepatocellular Carcinoma

Hepatocellular carcinoma (HCC) is one of the most prevalent malignancies worldwide. It has been found that GSDMD‐mediated pyroptosis and inflammation play an important role in pathological processes such as liver fibrosis [216]. The expression of GSDMD in HCC cells is upregulated, and tumor occurrence and progression in animal models can be inhibited by knocking out this gene [217]. Given the inhibitory effect of DMF on GSDMD, Lv and colleagues demonstrated its tumor suppressive effect in HCC, and combined treatment with anti‐PD‐1 would achieve better results [217]. They found that GSDMD not only inhibited IFN‐β release to promote tumor progression, but also induced STAT1 phosphorylation to upregulate PD‐L1 expression, which mediated immune evasion of tumors. Downregulation of prenyldiphosphate synthase subunit 2 (PDSS2), which is essential for the synthesis of CoQ10 and antioxidation, is an important driver of HCC tumorigenesis [218]. PDSS2–Del2, characterized by a deletion of exon 2, has an effect of promoting HCC metastasis and angiogenesis [219]. Elevated PDSS2–Del2 expression in HCC results in decreased fumarate levels, as well as activation of the canonical NF‐κB, Wnt/β‐catenin pathways [219]. By oral administration of DMF, metastasis of HCC cells can be inhibited, and inhibition of the NF‐κB pathway can partially explain this phenomenon [219].

#### 4.5.4 Lymphoma

Currently, NF‐κB inhibitors perform satisfactorily in the treatment of hematologic malignancies, such as acute myeloid leukemia and B‐cell lymphoma, which are dependent on NF‐κB activity [34, 220]. Tsubaki and colleagues found that DMF induced apoptosis in a variety of hematopoietic tumor cells, which was attributed to restricted NF‐κB nuclear translocation and subsequent reduced expression of Bcl‐xL and XIAP and Caspase‐3 activation [221]. Interestingly, normal lymphocytes were not affected, which could be explained by differences in the activity of NF‐κB. Subsequent studies also found that DMF effectively inhibited the growth and metastasis of cutaneous T‐cell lymphomas by inhibiting NF‐κB [111, 112]. With the deepening of research, the phenomenon and mechanism of DMF‐induced ferroptosis in cancer cells have also been gradually excavated. Schmitt and colleagues found that the GCB DLBCL cell line was susceptible to ferroptosis, possibly because GSH levels in such cells were lower and more susceptible to DMF than in other cells, such as the ABC DLBCL cell line [34]. Inhibition of NF‐κB pathway by DMF also contributes to the development of ferroptosis, as NF‐κB induces the expression of antioxidant proteins [34, 222]. They found that the use of bromodomain and extra‐terminal domain protein inhibitors could enhance the ferroptosis‐inducing effect of DMF [223].

#### 4.5.5 Other Cancers

In addition to the above several major cancers, fumarates also affect the occurrence and development of other cancers. Fumarates affect multiple cancers by targeting the same signaling pathway and also target numerous pathways in the same cancer. Consistent with inhibition of melanoma, DMF impairs the growth, proliferation, and metastasis of breast cancer cells primarily through inhibition of the NF‐κB signaling pathway [45, 114, 224]. A recent study has found that DMF can also covalently modify cysteine residues of ZNF217, a finger protein that can drive estrogen receptor‐positive breast cancer, to exert efficacy [225]. DMF showed a role in the proliferation of non‐small cell lung cancer cells in vitro and also successfully inhibited tumor progression in preclinical animal models [226]. We suggest that inhibition of NF‐κB by DMF may partially explain its inhibitory effect in this type of lung cancer [227]. There is currently a lack of studies on the possibility of MMF application in cancer treatment, perhaps because its reactivity with cysteine thiols is significantly weaker than that of DMF. However, Tavallai and colleagues provide us with new ideas [228]. They found that MMF combined with myelofibrosis drug ruxolitinib killed brain, breast, lung, and ovarian cancer cells. The combination of the two drugs modulated multiple signaling pathways, for instance, inhibiting the phosphorylation of STAT3/5, ERK1/2, AKT, and mTOR, decreasing BCL‐XL and MCL‐1 expression, and increasing ROS levels [228]. In summary, DMF has anticancer properties in a variety of tumors, but often requires high concentrations. As the study found, low concentrations of DMF have a protective effect on cells by activating the NRF2 pathway, while high concentrations of DMF are toxic to cells by depleting NRF2 through modification of DJ‐1 [150]. This once again shows that succination depends not only on the intrinsic properties of the electrophiles, but also on their concentration levels, which can lead to different target proteins being modified. Recently, Li and colleagues found that increasing fumarate levels by tyrosine supplementation could enhance chemosensitivity in epithelial ovarian cancer [229], and the role of fumarate in cancer deserves further study. Similarly, MMF combined with other drugs for cancer therapy is also a strategy that promises to reduce the side effects of drugs.

### 4.6 Clinical Trial Progress and Challenges of Fumarate and FAEs

Currently, clinical investigations into FAEs have gradually expanded from conventional immunomodulatory indications into multisystem disorders (Table 4), focusing primarily on their immunomodulatory, anti‐inflammatory, and neuroprotective potentials. Given that clinical trials specifically evaluating MMF remain scarce and its therapeutic spectrum is largely encompassed by research on DMF, including MS (NCT04022473, NCT04570670) and psoriasis (NCT02955693), the following discussion will focus on DMF. In the field of dermatology and immunology, clinical trials evaluating DMF have been conducted for chronic inflammatory diseases, including plaque psoriasis and rheumatoid arthritis. While DMF exhibited excellent therapeutic efficacy in patients with moderate‐to‐severe chronic plaque psoriasis, clinical benefits appeared less pronounced in those with active rheumatoid arthritis [230]. In the context of neurological disorders, research has predominantly centered on MS, primary progressive MS (PPMS), radiologically isolated syndrome, and adrenomyeloneuropathy (AMN), alongside investigations into its neuroprotective potential in acute ischemic stroke. Published data robustly support the neuroprotective effects of DMF; for instance, a clinical study confirmed that 12 months of DMF treatment significantly reduced neurofilament light chain levels in the serum, plasma, and cerebrospinal fluid of patients with RRMS [231]. However, a 12‐month intervention failed to yield clinical improvements in PPMS, with no parallel decline observed in cerebrospinal fluid neurofilament light chain levels. Furthermore, although DMF in combination with riluzole was reported to be safe and well tolerated for the treatment of amyotrophic lateral sclerosis, its therapeutic efficacy remained insufficient [232]. In the context of oncology and hematological malignancies, DMF has been deployed in early exploratory clinical trials for glioblastoma, cutaneous T‐cell lymphoma, and chronic lymphocytic leukemia, with research efforts heavily focused on evaluating its efficacy, safety, and tolerability in combination regimens. Furthermore, DMF is concurrently undergoing exploratory or early‐stage clinical validation for other conditions, including unruptured intracranial aneurysms, obstructive sleep apnea, Type 1 diabetes mellitus, and AMD. Collectively, the clinical research landscape of DMF exhibits a progressive expansion from conventional autoimmune diseases toward neuroprotection, anti‐inflammation, and the regulation of multipathological pathways. This broadening paradigm underscores its multitarget immunomodulatory characteristics and highlights its potential therapeutic value across a diverse spectrum of diseases.

**TABLE 4:** Summary of clinical or preclinical studies of DMF.

Disease	Location countries	Phase	Last known status	Objectives	Preliminary findings	NCT number
Plaque psoriasis	Austria and other countries	Phase 3	Completed	To evaluate the efficacy and safety of LAS41008 (DMF) versus active control and placebo in patients with moderate‐to‐severe chronic plaque psoriasis	DMF is effective in the treatment of adults with moderate‐to‐severe chronic plaque psoriasis.	NCT01726933
Rheumatoid arthritis (RA)	Australia and other countries	Phase 2	Completed	To evaluate the efficacy and safety of DMF in patients with RA who have an inadequate response to methotrexate	NA	NCT00810836
Primary progressive multiple sclerosis (PPMS)	Denmark	Phase 2	Completed	To evaluate safety and efficacy of DMF treatment in patients with PPMS	48 weeks of DMF treatment did not reduce cerebrospinal fluid neurofilament light chain levels or show clear clinical or MRI benefits.	NCT02959658
Multiple sclerosis (MS)	United States and other countries	NA	Completed	To evaluate the long‐term safety and effectiveness of DMF in patients with MS	In real‐world clinical practice, DMF showed a safety profile consistent with its known adverse effect profile. Relapse rates were low, and both annualized relapse rate and patient‐reported outcomes remained stable over time.	NCT02047097
Radiologically isolated syndrome (RIS)	United States	Phase 4	Completed	To evaluate whether DMF can prevent the first clinical symptom in patients with RIS, the earliest preclinical stage of MS	DMF significantly reduced the risk of first clinical demyelinating event in RIS patients.	NCT02739542
Acute ischemic stroke (AIS)	China	Phase 2	Recruiting	To evaluate the safety and efficacy of combining DMF with alteplase for the treatment of AIS	DMF modulates peripheral and central immune responses, reduces infarct volume, promotes microglial recruitment, and preserves neuronal survival after ischemic stroke.	NCT04890366
Adrenomyeloneuropathy (AMN)	Spain	Phase 2 Phase 3	Active	To assess the efficacy of DMF for motor impairment in adults with AMN	NA	NCT06513533
Glioblastoma multiforme (GBM)	United States	Phase 1	Completed	To evaluate the safety, tolerability, and optimal dose of DMF in combination with temozolomide and radiotherapy for newly diagnosed GBM	DMF may be safely combined with radiation therapy and Temozolomide in patients with newly diagnosed GBM.	NCT02337426
Cutaneous T cell lymphoma (CTCL)	Germany	Phase 2	Completed	To evaluate the efficacy of DMF in patients with CTCL	DMF represents an effective and well‐tolerated therapeutic option for CTCL.	NCT02546440
Chronic lymphocytic leukemia (CLL)	United States	Phase 1	Terminated (lack of funding)	To evaluate the safety and tolerability of DMF in patients with relapsed/refractory CLL	NA	NCT02784834
COVID‐19	Belgium and other countries	Phase 3	Recruiting	To evaluate the efficacy of various treatment schemes for COVID‐19 inpatients	DMF provided no clinical benefit in hospitalized COVID‐19 patients when added to standard of care.	NCT04381936
Intracranial unruptured aneurysms	China	Phase 4	Not yet recruiting	To determine whether DMF could significantly reduce inflammation within the intracranial aneurysm wall	NA	NCT05959759
Obstructive sleep apnea (OSA)	United States	Phase 2	Completed	To evaluate whether oral DMF is an effective treatment for OSA in patients unable or unwilling to use positive airway pressure therapy	DMF is well tolerated and partially ameliorates OSA severity	NCT02438137
Type 1 diabetes	China	Phase 2 Phase 3	Recruiting	To evaluate the efficacy, safety, and tolerability of DMF in preserving pancreatic beta‐cell function in adults with Type 1 diabetes	NA	NCT07548996
Age‐related macular degeneration (AMD)	France	Phase 2	Recruiting	To assess the safety, tolerability, and preliminary efficacy of 12 months of DMF treatment in subjects with geographic atrophy associated with AMD	NA	NCT04292080

*Data sources*: clinicaltrials.gov.

Currently, the clinical application of FAEs faces several major challenges. First, although their mechanism of action involves NRF2 activation and immunomodulation, the pathological contribution of this pathway varies substantially across different diseases. Consequently, this relatively broad “anti‐inflammatory/antioxidant” mechanism fails to guarantee consistent therapeutic efficacy across diverse clinical indications. Second, the dose‐limiting toxicities of FAEs are primarily driven by adverse events such as gastrointestinal distress, flushing, and lymphopenia. This creates an inherent conflict between achieving effective systemic exposure and maintaining patient tolerance, thereby constraining the full realization of their therapeutic potential. Furthermore, in nonclassical indications beyond MS and psoriasis, the heightened disease heterogeneity and more intricate pathological mechanisms often lead to a decreased success rate in the clinical translation of single‐agent immunomodulatory strategies. Concurrently, several recent clinical trials yielding negative or inconclusive results suggest that the efficacy of FAEs in acute inflammation, the tumor microenvironment, or degenerative diseases may depend on specific patient subpopulations or early‐stage disease processes, rather than exerting a universal therapeutic effect. This collectively reflects a translational chasm during indication expansion, characterized as being “mechanistically feasible but clinically unpredictable.” Nevertheless, given that FAEs such as DMF are already regulatory approved, cost effective, and clinically well characterized, they remain highly attractive drug candidates for numerous diseases and warrant further clinical development.

## 5 Conclusions and Perspectives

### 5.1 Summary of Key Findings

As an important metabolic intermediate in the TCA cycle, fumarate plays a role in biological processes that go far beyond energy metabolism. There are similarities and differences between fumarate and FAEs represented by MMF and DMF in chemical properties and biological functions. This paper makes a systematic review and comparison.

As a crucial TCA cycle intermediate, fumarate is tightly maintained at low physiological levels under homeostasis. However, specific pathological conditions trigger aberrant fumarate accumulation, enabling it to act as a potent metabolic signaling molecule. In FH‐deficient tumors beyond HLRCC, FH deficiency directly drives massive fumarate accumulation, which in turn activates a cascade of oncogenic pathways and heightens cellular susceptibility to transforming oncogenic events [233]. Regarding the redox homeostasis network, elevated fumarate is intimately linked to increased ROS. Specifically, fumarate directly conjugates with GSH to form succinated GSH; this adduct consumes the reducing equivalents of NADPH via GSH reductase without exerting antioxidant capacity, thereby exacerbating mitochondrial ROS generation and stabilizing HIF‐1 [37]. Concurrently, fumarate‐mediated succination of KEAP1 triggers NRF2 nuclear translocation and upregulates antioxidant gene expression. This likely optimizes intracellular ROS levels, licensing cell proliferation and stress survival while averting cytotoxic damage. Metabolically, accumulated fumarate impairs HIF‐1α degradation via PHDs succination, fueling angiogenesis and glycolysis. Furthermore, fumarate drives tumorigenesis by instigating profound epigenetic remodeling. Beyond FH‐mutant malignancies, fumarate accumulation also intercepts immune and inflammatory responses. In LPS‐stimulated macrophages, rewiring of the TCA cycle dramatically elevates endogenous fumarate, while prolonged LPS exposure suppresses FH activity to amplify the Type I IFN response [15].

Second, succination is the primary mechanism through which fumarate and FAEs exert their effects. This modification may depend primarily on fumarate concentration, protein structure, and cellular environment, including pH and redox state. In terms of its own properties, the presence of diester groups makes DMF significantly more electrophilic than MMF and fumarate, which partly explains its stronger reactivity toward cysteine thiols. Consistently, DMF/MMF exposure can also lead to rapid depletion and subsequent recovery of intracellular GSH, reflecting its high electrophilic burden. Comparatively, fumarate alkyne exhibits reactivity most similar to endogenous fumarate, and may therefore be widely used to approximate physiological events [53]. Fumarate accumulation can also be achieved through genetic or pharmacological inhibition of FH, as observed in HLRCC or in experimental FH knockdown/knockout models. Notably, in HLRCC, the markedly acidic microenvironment may further promote fumarate protonation to form hydrogen fumarate, similar to MMF reactivity [234]. Unlike exogenous supplementation, FH loss leads to sustained intracellular fumarate accumulation within a complex metabolic context, often accompanied by broad rewiring of the TCA cycle, redox imbalance, and epigenetic alterations. Therefore, whether to use DMF, MMF, or other FAEs, or increase endogenous fumarate by FH inhibitor, FH knockout or other ways is still a consideration when studying the related effects.

Third, the pleiotropic effects of both fumarate and FAEs are largely governed by cell‐specific regulatory contexts rather than stochastic protein cysteine modification. We propose that the cellular metabolic background, antioxidant and electrophilic buffering capacities, and the baseline expression of target proteins and downstream genes are key determinants of this specificity. Distinct cell types and cellular states exhibit divergent expression profiles of metabolic enzymes, giving rise to substantial variations in metabolite composition and abundance. For instance, whereas tumor cells present an exceptionally high fumarate baseline due to FH deficiency, immune cells such as macrophages experience temporary surges in both fumarate and itaconate (which also reacts with cysteine sulfhydryl groups) driven by inflammation‐induced TCA cycle rewiring. Moreover, the regulatory efficacy of FAEs is intrinsically linked to the expression and activity of cellular esterases. Although oral DMF undergoes rapid systemic hydrolysis to MMF in vivo, MMF undeniably retains potent electrophilicity. The specific esterases involved in the hydrolysis of MMF to fumarate remain unidentified; variations in esterase expression levels and activity across different cell types may influence MMF levels and, consequently, its functional activity. Such differences in cellular regulation may be even more pronounced upon local administration of DMF. The intracellular antioxidant and electrophilic buffering capacity fundamentally modulates the biological outcomes of fumarate and FAEs. Deprotonated GSH represents one of the most efficient cellular nucleophiles [235]; consequently, the elevation of endogenous fumarate or the influx of exogenous FAEs invariably results in the initial depletion of the intracellular GSH pool. Neurons, characterized by low baseline GSH levels and a restricted de novo synthesis capacity, are exquisitely sensitive to GSH exhaustion [236]. They are thus highly vulnerable to the early‐stage GSH depletion triggered by FAEs, a liability that is subsequently counteracted by NRF2‐mediated cytoprotective responses. In contrast, immune cells possess robust GSH regeneration machinery; as a result, they actively undergo metabolic and immune reprogramming rather than succumbing to cytotoxicity upon fumarate accumulation or FAEs supplementation. In malignancies, a high baseline GSH pool coupled with exceptional regenerative capacity rapidly buffers electrophilic insults, suggesting that combining FAEs with other therapeutic agents holds greater promise for inducing cancer cell death. Ultimately, while the KEAP1–NRF2 axis stands as a master orchestrator of fumarate and FAEs signaling, the heterogeneous activation thresholds of NRF2 and the divergent expression patterns of its downstream target genes across various tissues and cell types dictate the final functional outcomes.

Fourth, fumarate‐targeted therapeutic interventions comprise two clinical vectors: targeting pathological fumarate accumulation and expanding FAEs applications. In FH‐deficient malignancies, where fumarate functions as an oncometabolite, leveraging synthetic lethality to selectively eliminate cancer cells is more effective than directly clearing fumarate, as FH loss induces complex cellular alterations beyond fumarate accumulation [237]. Currently, fumarate‐targeted therapy for HLRCC patients encompasses inhibitors targeting downstream effectors of HIF (e.g., the VEGF inhibitor bevacizumab paired with the EGFR inhibitor erlotinib), the ABL1‐targeted tyrosine kinase inhibitor vandetanib, and HMOX1 antagonists such as zinc protoporphyrin or imidazolium‐based derivatives like SLV‐11199 [237]. To uncover more efficacious interventions for these malignancies, experimental platforms involving small‐molecule FH inhibitors or genetic FH knockout models remain indispensable. On the other hand, the widespread clinical success of FAEs, particularly DMF and MMF, stems from their multifaceted immunomodulatory, antioxidant, and anti‐inflammatory properties. Future endeavors should focus on mapping their precise proteome‐wide targets to engineer next‐generation, highly selective fumarate‐based chemical entities. In this context, clickable chemical probes, mass spectrometry‐based chemical proteomics, and anti‐2SC antibodies will serve as pivotal discovery tools. Last, addressing tolerability issues and adverse side effects remains paramount. Current translational research is actively exploring innovative prodrug designs and localized targeted delivery systems to maximize regional therapeutic efficacy while minimizing systemic toxicity. Pinto et al. demonstrated that inhalation of DMF‐loaded solid lipid nanoparticles not only alleviated central nervous system inflammation and tissue damage in mice with experimental autoimmune encephalomyelitis but also protected them from pulmonary inflammation and functional impairment [238]. Similarly, Liu et al. designed a ROS‐responsive liposome for inhalable delivery of DMF, which provides a therapeutic strategy for idiopathic pulmonary fibrosis and other lung diseases [239]. In another study, Khosravi et al. designed nanoparticles loaded with DMF that target CD40 [240]. The use of anti‐CD40 monoclonal antibodies facilitates the targeted delivery of DMF to CD40‐expressing cells such as B cells, macrophages, and DCs. Compared with free DMF or DMF‐loaded nanoparticles without the use of anti‐CD40 antibodies, these nanoparticles demonstrated a superior ability to inhibit the expression of TNF‐α, IL‐1β, and IL‐6 in mouse splenocytes [240]. Although DMF has shown potential therapeutic effects in gastrointestinal tumors, its easy hydrolysis may hinder its application. The stronger cysteine thiol reactivity of DMF than MMF is an important reason for the difference in their biological activities in tumors, which is mainly due to the stronger electrophilicity brought by the diester group. Ma et al. developed an innovative delivery system involving DMF‐loaded azo polymeric micelles for targeted drug delivery to the colon [241]. This design leverages the specific secretion of azoreductase by colonic bacteria, which cleaves the azo bonds that connect olsalazine, a 5‐aminosalicylic acid prodrug. Upon reaching the colon, the azo bonds are cleaved by azoreductase, allowing for the localized release of DMF and 5‐aminosalicylic acid [241].

### 5.2 Technical Bottlenecks and Future Perspectives

Based on the above in‐depth analysis, we have identified several issues of significant clinical importance that must be addressed, as they are crucial for advancing the translational application of therapies based on fumaric acid:

1. The mechanism by which fumarate is transported between different cellular compartments and between inside and outside the cell remains to be elucidated. Addressing this question could provide insights into the metabolic reprogramming of FH‐deficient cells and clarify the role of fumarate as a signaling molecule, thereby offering clues for metabolic targeting therapies in diseases. If we can interfere with fumarate export pathways, it might mitigate its impact on other cellular compartments and the extracellular environment, thus inhibiting disease progression. Clarifying this issue could also help promote the entry of exogenous fumarate into cells for therapeutic effects.
2. It has been reported that miR‐144 expression is elevated in the liver of obese mice, which activates FH, depleting fumarate and impairing the antioxidant response [242]. Are there other noncoding RNAs that directly or indirectly regulate FH activity, thus controlling fumarate levels and affecting physiological or pathological processes? Investigating how noncoding RNAs regulate FH activity and fumarate metabolism could uncover potential therapeutic targets and provide a theoretical basis for developing new RNA‐based drugs or strategies.
3. Cysteine is arguably the most reactive amino acid in the human body. What is the link between succination and other ways of cysteine modification, such as oxidative modification and S‐nitrosation, and what are the consequences? Does fumarate cause other protein modification changes? There are likely interactions between cysteine succination and other modifications, which may be a critical factor in explaining why fumarate and FAEs exert different effects across various diseases. Furthermore, clarifying how fumarate and FAEs influence other types of protein modifications and the resulting biological consequences could facilitate more in‐depth research and contribute to developing more effective disease treatments.
4. How can the pharmacokinetics and pharmacodynamics of FAE‐based drugs be optimized to enhance efficacy and reduce side effects in clinical settings? The route of drug administration significantly impacts the absorption rate, bioavailability, and first‐pass metabolism of a drug. The combined use of nanocarriers, liposomes, and sustained‐release formulations can significantly improve drug stability, absorption, and distribution. Currently, oral administration of FAEs is the preferred option in most cases, and optimizing delivery strategies holds considerable clinical significance. Ongoing research should focus on developing safe, convenient, and cost‐effective delivery methods. Additionally, improving the targeting specificity of FAEs and exploring combination therapy strategies are essential for future research.

### 5.3 Concluding Remarks

This review has comprehensively discussed fumarate metabolism, its pleiotropic biological effects, its roles in pathophysiological processes, and associated therapeutic strategies. Given their close interrelationship, we have also reviewed and compared fumarate, FH, and FAEs. Undoubtedly, fumarate and its derivatives play crucial roles in maintaining human health and treating diseases. However, as outlined above, several foundational and clinical issues remain to be addressed. Resolving these questions will not only deepen our understanding of fumarate biology but also pave the way for more precise and effective clinical applications of fumarate‐based therapeutics.

## Author Contributions

PZ, MY, RR, and XX conceived the manuscript. PZ and MY reviewed the literature and wrote the original draft. PZ and MY generated the figures and tables. RR, YZ, JZ, and SC polished the manuscript. XX and RR reviewed and edited the final manuscript. All authors contributed to the article and approved the submitted version.

## Funding

This work was supported by the grants from the National Natural Science Foundation of China (No. 82401261), the National Key Research and Development Program of China (2024YFA1108700 and 2024YFA1108704), the China National Postdoctoral Program for Innovative Talents (BX20230434), the China Postdoctoral Science Foundation (No. 2023M743952), the Youth Science Foundation of Xiangya Hospital (No. 2023Q12), and the National Clinical Key Specialty of Ophthalmology.

## Ethics Statement

The authors have nothing to report.

## Conflicts of Interest

The authors declare no conflicts of interest.

## Data Availability

The authors have nothing to report.
